# Planting Season Impacts Sugarcane Stem Development, Secondary Metabolite Levels, and Natural Antisense Transcription

**DOI:** 10.3390/cells10123451

**Published:** 2021-12-08

**Authors:** Maryke Wijma, Carolina Gimiliani Lembke, Augusto Lima Diniz, Luciane Santini, Leonardo Zambotti-Villela, Pio Colepicolo, Monalisa Sampaio Carneiro, Glaucia Mendes Souza

**Affiliations:** 1 Departamento de Bioquímica, Instituto de Química, Universidade de São Paulo, São Paulo 05508-900, Brazil; maryke.wijma@alumni.usp.br (M.W.); clembke@iq.usp.br (C.G.L.); augustold@usp.br (A.L.D.); luciane.santini@usp.br (L.S.); lzv@iq.usp.br (L.Z.-V.); piocolep@iq.usp.br (P.C.); 2 Centro de Ciências Agrárias, Departamento de Biotecnologia e Produção Vegetal e Animal, Universidade Federal de São Carlos, São Paulo 13600-970, Brazil; monalisa@ufscar.br

**Keywords:** sugarcane, SP80-3280, metabolomics, HPLC-MS, transcriptomics, oligoarray, systems biology, multi-omics integration, secondary metabolism, antisense expression

## Abstract

To reduce the potentially irreversible environmental impacts caused by fossil fuels, the use of renewable energy sources must be increased on a global scale. One promising source of biomass and bioenergy is sugarcane. The study of this crop’s development in different planting seasons can aid in successfully cultivating it in global climate change scenarios. The sugarcane variety SP80-3280 was field grown under two planting seasons with different climatic conditions. A systems biology approach was taken to study the changes on physiological, morphological, agrotechnological, transcriptomics, and metabolomics levels in the leaf +1, and immature, intermediate and mature internodes. Most of the variation found within the transcriptomics and metabolomics profiles is attributed to the differences among the distinct tissues. However, the integration of both transcriptomics and metabolomics data highlighted three main metabolic categories as the principal sources of variation across tissues: amino acid metabolism, biosynthesis of secondary metabolites, and xenobiotics biodegradation and metabolism. Differences in ripening and metabolite levels mainly in leaves and mature internodes may reflect the impact of contrasting environmental conditions on sugarcane development. In general, the same metabolites are found in mature internodes from both “one-year” and “one-and-a-half-year sugarcane”, however, some metabolites (i.e., phenylpropanoids with economic value) and natural antisense transcript expression are only detected in the leaves of “one-year” sugarcane.

## 1. Introduction

The continuous use and misuse of fossil fuels have led to the risk of their depletion, as well as severe and potentially irreversible environmental threats [1,2]. Critical reductions in global greenhouse gas (GHG) emissions are required to achieve the goal of keeping the increase in the average global temperature to well below 2 °C in the context of sustainable development and poverty eradication [3]. One way of achieving this is to increase the use of renewable energy sources, such as biofuels, like bioethanol produced from sugarcane, which can be used to fuel society as well as reduce GHG emissions [4]. Significant alterations in climatic patterns are being observed due to global climate change and its effects are expected to increase even more by the turn of the century [5].

In Brazil, sugarcane plants are mainly planted in two different seasons (January to March or October/November) in South-Eastern Brazil and the cropping season and best suited varieties vary for each [6]. When planting is carried out between September and early December, the term “one-year” sugarcane is used since the crops are harvested around 12 months after planting. These crops present productivity estimates of less than 100 t/ha. On the other hand, when planting is carried out between early January or April, the crops are grown for a longer period of time hence the name “one-and-a-half-year” sugarcane, and present higher yields [7]. These higher yields are attributed to the longer growing periods. The lower air temperatures combined with moderate water deficit are the major ripening factors that result in an increase in sucrose content in sugarcane stalks. The planting date has a higher effect on sugarcane yield rather than on ripening (Pol levels) [7]. The mechanisms that regulate sugarcane development and the shifts between the different phenological stages still require more in-depth research.

During plant development, plants respond to developmental and environmental signals in their internal and external environments by relying on the activity of a variety of cellular components, on gene expression, and on metabolite (small molecules of size < 1800 Da) regulation, that trigger the appropriate metabolic responses needed for survival [8,9,10] and are the basis of crop yield and quality [11]. One of the mechanisms of gene expression regulation is through natural antisense transcripts (NATs) that are also regulated by different conditions [12].

Taking a systems biology approach when integrating metabolomics data with gene expression information and conducting in-depth data mining, it is possible to find connections between specific traits, the genes that code for them, and the metabolites and/or metabolic pathways that play a role in the regulation of important agronomic and economically valuable processes [13]. In addition, important conclusions regarding carbon partitioning can be drawn and value-added products can be identified which could be used by other industries and biorefineries. The current method of choice for conducting untargeted metabolomics studies is liquid chromatography coupled to high resolution mass spectrometry (LC-MS) due to its high throughput, soft ionization, and good coverage of a wide range of chemically diverse metabolites [14]. SP80-3280 was chosen for this study as it ranks among the top 20 sugarcane varieties grown in the state of São Paulo, Brazil, is being used as a genitor in Brazilian breeding programs [15] and a copy-resolved assembled gene space of this variety is available [16], thus making it the optimal choice for the exploration of the sugarcane metabolome, transcriptome, and their integration in a systems biology approach.

## 2. Materials and Methods

### 2.1. Plant Material and Experimental Design

The commercial sugarcane variety SP80-3280 was planted in two different climatic conditions at the Agricultural Science Center of the Federal University of São Carlos (UFSCar) in Araras City, São Paulo State, Brazil. For this purpose, two experimental fields were prepared with eutroferric oxisol soil fertilized with 400 kg ha-1 of N:P:K mixed in a ratio of 5:25:25. Each field experiment consisted of eight plots (10 m × 3 m) in which the sugarcane billets were planted in four rows of 10 m with 1.35 m spaces between the furrows in each plot. The first field experiment (F1) was initiated in April 2012 and extended up until May 2013, representing the conditions under which “one-and-a-half-year” sugarcane crops are cultivated. The second field experiment (F2) with the same technical specifications was initiated in October 2012 and extended up until November 2013, representing the conditions under which “one-year” sugarcane crops are cultivated. No irrigation was applied in this study and during these periods the plants experienced variations in the climatic conditions with regards to precipitation and temperature. Tissue samples were collected from the two middle rows of the plots from both fields at 4, 8, 11 and 13 months after planting for metabolomics and transcriptomics studies. Samples were collected from the uppermost visible dewlap leaves, referring to them as the Leaf +1 (L1); the immature internodes near the apical meristem, referring to them as I1; the internodes 5 which are considered intermediate internodes, referring to them as I5; and the internodes 9 which are considered mature internodes, counted from the first immature internode (I1) and referring to them as I9, as described in Papini Terzi et al., 2009 [17].

The effect of temperature variations on sugarcane development through the two conducted essays was investigated by the accumulated growing degree days (*AGDD*) analysis, calculated by the following equation:

(1) $$AGDD=\overset{n-1}{\underset{i=1}{\sum }}(Ta_{i}-Tb_{})$$

where *Ta_i_* is the average temperature (°C) on the *i*^th^ day and *Tb* is the base temperature (°C) for sugarcane development. The Tb considered here was suggested in [18] for all sugarcane processes and phenological phases (8 °C). The *AGDD* at each sampling point was calculated by temperature recorded until the day before sampling (*n − 1*).

### 2.2. Morphological, Technological and Physiological Data Measurements

A 10-stalk sample was taken for analysis of the stalk width (mm), stalk height (m), stalk number (SN), and number of internodes [19]. Using these stalks, technological analyzes were carried out such as BRIX (BRIX, in °Brix), sucrose content of the cane (POL%C, in %), sucrose content of the juice (POL%J, in %), and Juice Purity (Pza, in %) [20]. The soluble carbohydrates of the lyophilized leaves (200 mg) was determined using the phenol-sulfuric acid method as described [21]. The photosynthetic rate, transpiration rate, and stomatal conductance were measured with the LCiPro photosynthesis system with an LED light source at Q1500 (ADC Bioscientific, Hoddesdon, United Kingdom).

### 2.3. Transcriptomic Analysis

#### 2.3.1. RNA Extraction and Oligoarray Analysis

Total RNA extraction was done using TRIzol Invitrogen™ and grinded frozen sugarcane tissues according to the manufacturer’s instructions. Total RNA was treated with DNase I (Invitrogen™, Waltham, MA, USA) followed by purification with the RNeasy mini kit (Qiagen™, Hilden, Germany). RNA integrity and concentration were evaluated using a NanoDrop 1000 spectrophotometer (Thermo Scientific™, Waltham, MA, USA) and 200 ng of total RNA was used for labeled cRNA synthesis. For oligoarray analysis, the custom 4 × 44 k Agilent™ sugarcane oligonucleotide array was used, and sample preparation and hybridizations were performed as per Lembke et al. (2012) and Agilent™ protocols provided with the following kits: Quick Amp Two-Colour Labelling Kits (Agilent™, Santa Clara, CA, USA), Gene Expression Hybridization Kit, and Gene Expression Wash Buffer Kit. The RNeasy Mini Kit (Qiagen™) was used again for purification. For each sampling point, four biological replicates (two from each plot) and dye swaps were used (Table S1). For the leaves, a pool of all leaves and for the internodes, a pool of all internodes were used in the hybridizations. After overnight hybridization, the washed slides were scanned using a GenePix 4000B scanner (Molecular Devices™, San Jose, CA, USA) and the data was extracted using the Feature Extraction 9.5.3.1 software (Agilent™). The extracted data was uploaded onto the SUCEST-FUN database for automatic data analysis with the Cane Gene Expression tool (http://sucest-fun.org/wsapp/, accessed on 23 October 2015). Normalization was performed using non-linear LOWESS normalization [22] to correct for intensity-dependent dye.

#### 2.3.2. Multidimensional Scaling (MDS), Heatmap, GO Terms Enrichment, and Differentially Expressed Genes

The significantly expressed transcripts, both sense and antisense, from all replicates from all tissues, sampling points, and fields, were selected via the SUCEST-FUN database to be used for MDS analyses using the DGEList and plotMDS functions implemented in the “edgeR” R package [23].

Differentially expressed genes (DEGs) were estimated by functions implemented in the “limma” R package [24]. Background corrections and normalizations were applied from the “backgroundCorrect” and “normalizeWithinArrays” functions, respectively.

Heatmaps were constructed with the 500 most variable genes within the datasets. Specific gene clusters were detected, and gene set enrichment analyses (GSEA) were conducted using the “topGO” R package with the respective gene sets in each cluster to determine the top gene ontology (GO) terms related to each cluster [25].

### 2.4. Untargeted Metabolomics Analyses

#### 2.4.1. Samples Used and Global Metabolite Extractions

The samples were prepared by using eight biological replicates of each tissue (L1, I1, I5, I9) and sampling points (C1, C2, C3 and C4), that is four replicates from one plot and four from another plot. Pools of two samples each were made to reduce within-sample variation, rendering four biological replicates. The frozen samples were homogenized and aliquots of approximately 50 mg tissue powder were used for global metabolite extraction using the extraction method proposed by Salem et al. (2016) [26] with methyl tert-butyl ether (MTBE):methanol (MeOH) mixed in the ratio 3:1 and kept at −20 °C, followed by the addition of 650 μL of a H_2_O:MeOH solution mixed in the ratio 3:1 and kept at 4 °C. The H_2_O:MeOH phase from each sample was collected and placed in 2 mL microtubes in which they were stored at −80 °C prior to the HPLC-MS analysis. The pellet of each tube was dried down using speedvac for 1 h at 60 °C and weighed again for normalization purposes.

#### 2.4.2. HPLC-MS Analysis, Data Acquisition, Data Processing, and Metabolite Identification

In order to separate, ionize, and detect the polar and semi-polar metabolites in each sample, high-performance liquid chromatography (UFLC, Shimadzu, Kyoto, Japan) coupled to the high-resolution time-of-flight mass spectrometer (Bruker Daltonics, Billerica, MA, USA) was implemented. A pentafluorophenyl propyl ligand column (Phenomenex Luna PFP, 100 mm × 4.60 mm × 2.6 μm) was coupled to an ESI-microTOF mass spectrometer, operating in the positive and negative ionization modes. Mobile phase A (MPA) and Mobile phase B (MPB) used for compound separation and detection in the positive ionization mode were 0.1% formic acid (FA) in H_2_O with a pH of 2.65 (MPA), and MPA:MeOH:acetonitrile mixed in the ratio 5:25:70 (MPB). On the other hand, MPA and MPB used for compound separation and detection in the negative ionization mode were 20 mM NH_4_Ac in water with a pH of 7.35 (MPA) and MPA:MeOH mixed in the ratio 5:95 (MPB). A volume of 200 μL of each sample was transferred to a glass insert inside a glass vial prior to the analysis. A volume of 10 μL was injected into the HPLC-MS system and the flow rate was set at 500 μL/min. Gradient runs were conducted as in Table S2.

The raw HPLC-MS data were extracted and converted to the mzXML file format after which they were separated based on tissue type to prevent possible matrix effects since the four tissues are anatomically and metabolically different. The tools and packages used here were all implemented using the R-software. The XCMS (various forms (X) of chromatography mass spectrometry) package was used for pre-processing of the HPLC-MS data (peak filtration and identification, baseline correction, matching the peaks across the different samples, correcting the deviation in retention times, and filling in the missing peak information) [27].

The CAMERA package (Collection of Algorithms for Metabolite Profile Annotation) was used for ion, adduct and fragment annotation [28]. The outputs generated were then used as inputs for ProbMetab to predict the most likely compounds present in each tissue, thus allowing for a more robust and accurate metabolic profile of each tissue collected [29]. This package allowed direct links to the Kyoto Encyclopedia of Genes and Genomes (KEGG) database, mapping each metabolite to their respective pathways. The intensity of each predicted metabolite was normalized to the dry weight (DW) of the sample and selected for the downstream analyses.

Raw metabolomics data require pre-processing, in this case via the use of XCMS, CAMERA and ProbMetab, in order to generate “clean” data, which were the inputs for the data pre-treatment steps. Data was normalized by dry weight (DW), log10 transformed and no scaling technique was needed to be applied to yield optimal results for the untargeted metabolomics data based on Shapiro–Wilk’s normality tests and PCAs.

The tissues were then separated to identify metabolic differences observed in each tissue between the plants that were planted in different seasons of the year (field comparisons). The same discriminant analyses were thus conducted namely PCA and PLS-DA, as described.

### 2.5. The Integration between Two Data Modalities: Metabolomics and Transcriptomics

#### 2.5.1. Metabolic Pathway Activities

After the raw HPLC-MS data was generated and processed, the KEGG metabolites predicted by ProbMetab and their normalized, transformed, and scaled intensities in each sample were subjected to pathway activity profiling (PAPi) [30]. This R package was used to calculate the KEGG pathway activity scores in each tissue over time, which allowed us to observe how the identified pathways change between the different conditions throughout development (across sampling comparisons). The pathway activities of selected pathways were plotted using MetaboAnalyst 4.0 [31,32].

#### 2.5.2. The Implementation of the Multi-Omics Factor Analysis (MOFA) Tool

In order to determine which and where variation is shared between the two data modalities represented in this study, namely metabolomics and transcriptomics data, MOFA [33] was implemented using the output results from PAPi and the significantly expressed transcripts involved in the respective activated pathways to highlight which and how the metabolic pathways are altered throughout sugarcane development in the field.

The sequences of the significantly expressed transcripts from the activated metabolic pathways identified by PAPi were obtained from the SUCEST-FUN database (http://sucest-fun.org/wsapp Accessed on 14 August 2019). The transcript sequences were used as an input for blast2GO [34] analyses to retrieve the enzyme codes (E.C.) and to annotate and assign each significantly expressed transcript to the KEGG database. Only the annotated transcripts which matched to at least one KEGG enzyme on at least one of the activated metabolic pathway maps were selected for downstream analyses for the integration between the metabolomics and transcriptomics data modalities using the MOFA tool [33].

All factors, in this case, the activated metabolic pathways and the selected significantly expressed transcripts explaining less than 1% of the variation in both metabolomics and transcriptomics data were excluded from the analysis as per default settings and advice from the creator of MOFA [33]. The latent factors (LFs) with the most shared variation between the two data modalities were investigated by selecting the top 10 metabolic pathways and the top 300 significantly expressed transcripts shared within each LF. The 300 significantly expressed transcripts were used as an input for GSEA using the topGO R package to get the top 10 GO terms related to the respective transcripts [25].

### 2.6. Statistical Analysis

Morphological, technological and physiological data was analyzed as a split-plot scheme in a balanced Completely Randomized Design (split2.crd). Analysis of variance (ANOVA) and Fisher’s least significant difference (LSD) tests were conducted. When the interaction between the two factors (F1 and F2) was not significant, the data were analyzed as completely randomized design under one single factor. The analysis was done using ExpDes R Package [35].

The significantly expressed transcripts from the oligoarray analysis were identified as described in Lembke et al., 2012 [36]. Briefly, it is based on the significance test of the feature extraction software which uses the local background signal and the Background-subtracted green signal in both biological replicates. As described above, “limma” R package was used for DEGs identification.

For the untargeted metabolomics, the ProbMetab R package was used for most likely compound estimation [29]. It uses a Bayesian model to provide a list of compound candidates ranked by their respective probabilities. This package was developed specifically for the annotation of LC-MS data by using a set of known chemical reactions between candidate compounds since combinations which are detected together would make more biochemical sense than others. The proposed masses that matched to the possible metabolites from the ProbMetab output with the highest p-values were then chosen as the most likely metabolites for downstream analyses. The identification parameters and the metabolite identification results from each anatomical tissue and both positive and negative ionization modes are shown in Supplementary Data S1. Once the optimal metabolomics datasets (processed, normalized, transformed, and scaled metabolomics datasets) were obtained, they were used as inputs for the construction of PCA and partial least squares discriminant analysis (PLS-DA) models in MetaboAnalyst 4.0. The models were investigated, and from the PLS-DA models, the metabolites identified with VIP scores above 1 were selected for generating heatmaps in MetaboAnalyst 4.0 [37] to highlight the main contributors of the observed separations. This was done in order to determine the main metabolic differences between the four different tissues analyzed (tissue comparisons).

## 3. Results

**Figure 1:**
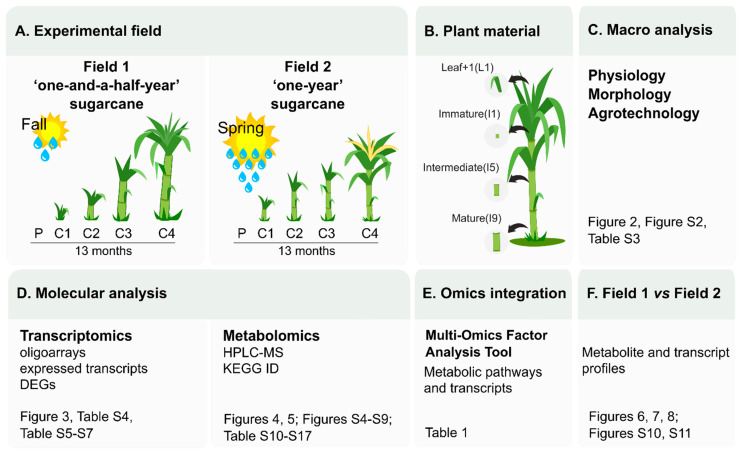
Experimental design and guide to datasets. (**A**) The sugarcane variety SP80-3280 was field grown under two planting seasons (Fall and Spring) with different climatic conditions and data and sample collection were done after 4 (C1), 8 (C2), 11 (C3) and 13 (C4) months after planting (P). (**B**) Plant material collected for molecular analysis: leaf +1 (L1), immature (I1), intermediate (I5) and mature internodes. A systems biology approach was taken to study the changes on (**C**) physiological, morphological, agrotechnological, (**D**) transcriptomics, and metabolomics levels. (**E**) The MOFA tool was used to point out the main metabolic pathways that were activated and altered throughout development. (**F**) Finally, comparisons between the results from Field 1 and Field 2 were done in each sugarcane tissue studied.

A summary of the experimental design and a guide to the results and datasets is presented in Figure 1.

### 3.1. Different Climatic Conditions Influence Sugarcane Development and Ripening

**Figure 2:**
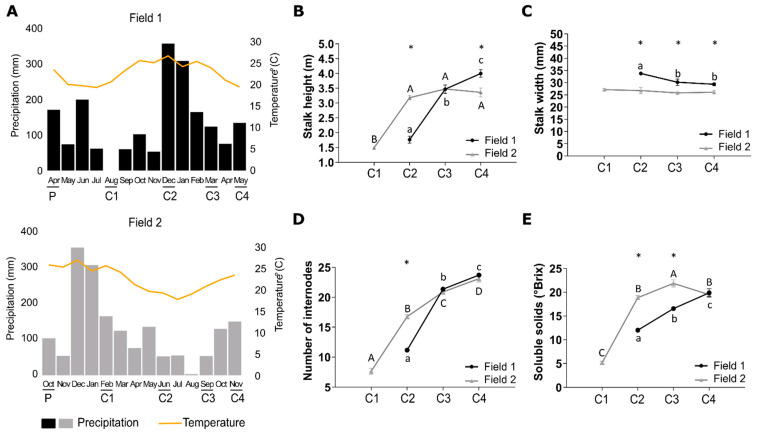
Precipitation and temperature during the initial months after sugarcane planting affect initial sugarcane elongation and stalk height, width, and soluble solids content at the stage of ripening. (**A**) Precipitation and temperature measures of fields 1 (F1) and 2 (F2). (**B**) Stalk-height, (**C**) stalk width, (**D**) number of internodes, and (**E**) soluble solids content in F1 and F2 in 4 (C1), 8 (C2), 11 (C3), and 13 (C4)-month-old plants. Letters and * are from the split2.crd results. Means with the same letter are not significantly different and means with different letters are significantly different between collection points. * shows differences between F1 and F2. Error bars show the standard error of the mean.

The plantings of SP80-3280 were carried out in two seasons of the year and with different climatic conditions which are common for sugarcane cultivation, and are classified as “one-and-a-half-year” sugarcane (F1) and “one-year” sugarcane (F2), respectively (Figure 2A).

The sugarcane plants from F1 experienced an initial growth period under low average temperature and precipitation, whereas the plants from F2 experienced an initial growth period under high average temperature and precipitation (Figure 2A). These different climatic conditions led to different developmental profiles and morphologies of the plants from the two different fields. The late development of plants in the initial stages at F1 is corroborated by the differences between the Accumulated Growing Degree Days (AGDD) values obtained in both fields (Figure S1). At the first sampling point (C1), the AGDD was 1.511 °C in F1 and 2.336 °C in F2, a difference of 824 °C between both time points. This was the higher difference of AGDD between both fields and coincides with the higher difference in the plants’ phenological stages as in C1 the plants from F1 presented only leaves while the plants from F2 presented leaves and stalks. At 11 and 13 months after planting (C3 and C4, respectively), the AGDD values were higher in F1 and the plant height in this field was higher at 13 months (C4) (Figure 2 and Figure S1).

Instantaneous photosynthetic rate, transpiration rate, stomatal conductance and water use efficiency were similar in the two fields (Figure S2). The plants from F1 presented retarded vegetative growth and elongation during the initial four months after planting since no internodes were formed at the first sampling (C1), thus no measurements of the height and the number of internodes could be taken (Figure 2). At the second sampling (C2) of F1, internodes were visible indicating that the vegetative growth and elongation phase had been initiated after which the plants continued to grow and elongate, even following the third (C3) and fourth (C4) sampling (black bars in Figure 2B). The ripening phase started at C4 when Pol%juice, Pol%cane, Purity%, and °Brix% reached values of 15.50, 13.20, 84.93, and 19.87, respectively (Table S3) [38,39] but the plants did not reach full maturation in the studied period as °Brix values continued to increase (Figure 2E). The sugarcane plants from F2 presented a rapid vegetative growth and elongation phase during the initial four months since internodes were already visible at C1 and the height of the plants could be measured at this stage (grey bars in Figure 2B). At C2, C3, and C4 it was observed that this growth and elongation phase had stabilized between eight and eleven months after planting since no increases in plant height and in the number of internodes were observed throughout C2 to C4. The ripening occurred at C3) when Pol%juice, Pol%cane, Purity%, and Brix% reached values of 19.64, 16.75, 90.71, and 21.86, respectively (Table S3).

Comparing the plants from the two different fields to each other at C2 (8 months), it was evident that the sugarcane plants from F2 were significantly taller, had more internodes, and presented a narrower culm diameter which persisted throughout development (Figure 2B–D) which showed that plants from the two fields were in different phenological stages. However, at C4 the plants from F1 had significantly surpassed the height and number of internodes as compared to those from F2 (Figure 2B), suggesting that they extended the vegetative growth and elongation phase, whereas the plants from F2 experienced the shift between this phase and the ripening phase between C2 and C3. These findings were also supported by analyzing the changes in the Brix content over time which shows that soluble solids were accumulated more rapidly in plants from F2 (Figure 2E). The plants from F1 presented a continuous significant increase in the Brix content over time, however, it was always lower than the plants from F2 in which the Brix content had reached its maximum at C3 after which it decreased again when flowering occurred (Table S3).

Sucrose contents increased from leaf tissues to immature internodes all the way down to the mature internodes. The biggest difference between sucrose content in I5 and I9 was at C4. The I9 from the plants from F2 presented higher sucrose content than the I9 from F1 at all sampling points (Figure S3).

### 3.2. Most of the Variation within Transcriptomics Profiles Is Attributed to the Differences between the Distinct Anatomical Tissues

**Figure 3:**
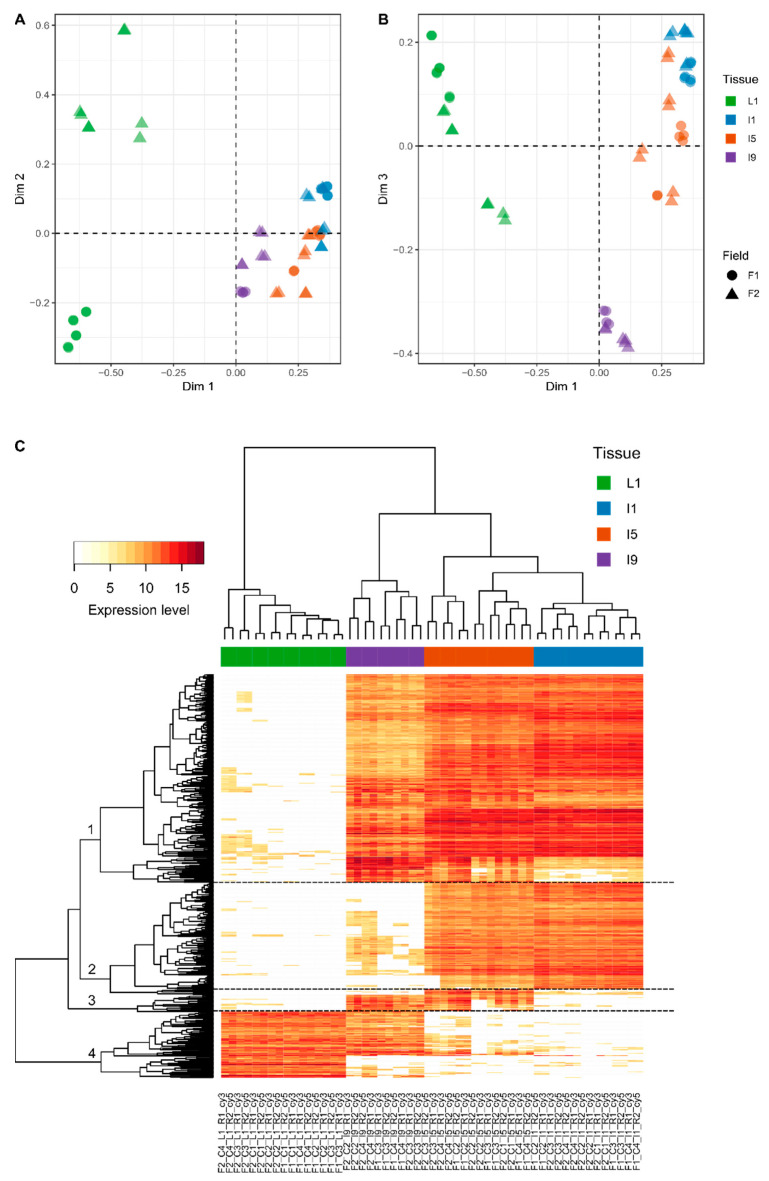
Multidimensional scaling (MDS) analyses of the transcriptomics data demonstrated that most of the variation within transcriptomics profiles is attributed to the differences between the distinct anatomical tissues. (**A**) Dim1 vs. Dim2; (**B**) Dim1 vs. Dim3. (**C**) Heatmap constructed using the top 500 most variable genes responsible for the variations in the different tissues. L1, I1, I5, and I9 refer to leaf +1 and immature, intermediate, and mature internodes. F1 and F2 refer to the plants that were planted in April 2012 and October 2012, respectively. C1, C2, C3, and C4 refer to sampling points at 4, 8, 11, and 13 months after planting.

Out of the 21,902 different probes (14,522 Sugarcane Assembled Sequences, SAS) represented on the CaneRegNet oligoarray, 15,552 (13,595 sense and 4091 antisense) were significantly expressed when all tissues, sampling points, and field conditions were considered (Table S4). Multidimensional scaling (MDS) analyses using these transcripts evidenced a distinct expression pattern between the different tissues (L1, I1, I5, and I9), represented mainly by the first dimension (Figure 3A,B). The second dimension allowed for the distinction between the different experimental conditions (F1 and F2) in the L1 and I9 tissues, respectively.

In addition, the third dimension contributed to a clear distinction between I9 and the remainder of the tissues (Figure 3B). Heatmap analysis of the 500 most variable genes within the dataset allowed, in addition to the clear distinction between the tissues, the identification of four specific expression patterns (Figure 3C) regardless of the sampling point, highlighting yet again that the main differences exist due to the different anatomical tissues and their specified metabolisms. The first cluster included 258 genes whose expressions were mostly absent in L1. This gene set was enriched for genes with GO terms associated with carbohydrate metabolism, specifically cell wall organization and construction (Table S5). The second cluster was composed of 139 genes whose expressions were higher in I1 and I5 and were enriched for GO terms related to the cell cycle, including chromosome organization and DNA replication (Table S5). The third cluster presented 21 genes mostly expressed in I5 and I9 and enriched for GO terms related to the development of conducting vessels (xylem and phloem) and lignin metabolism (Table S5). Finally, the fourth cluster included 82 genes whose expressions were higher in L1 and the top GO term pointed towards secondary metabolism (Table S5). The number of differentially expressed genes (DEGs) in each tissue along its development decreases with sugarcane maturation (Table S6). For example, L1 from 4- and 8-month-old plants have 645 DEGs whereas L1 from 11- and 13-month-old plants have only 71 DEGs in F1. Internode 9 from 8- and 11-month-old plants have 212 DEGs whereas I9 from 11- and 13-month-old plants have only 62 DEGs in F2. The only exception of this pattern is I5 from F2 in which 307 DEGs were identified from 8- and 11-month-old plants and only 7 DEGs from 11- and 13-month-old plants. The highest number of DEGs between F1 and F2 was observed in L1 (1104 DEGs) (Table S7).

### 3.3. Most of the Variation within Metabolomic Profiles Is Attributed to the Differences between the Distinct Anatomical Tissues

In total, 108 samples consisting of L1, I1, I5, and I9 tissues were used for HPLC-MS analyses and the acquisition of untargeted metabolomics data. After feature detection, nonlinear retention time alignment, and relative quantification across all samples, hundreds of metabolic features were detected in both positive and negative ionization modes. The data were normalized by dry weight (DW), log10 transformed, and no scaling technique was needed to be applied to yield optimal results for the untargeted metabolomics data based on Shapiro–Wilk’s normality tests and PCAs (Tables S8–S10). In the four different tissues analyzed here, 90, 84, 79, and 54 metabolites with unique KEGG IDs were predicted (Tables S11–S15).

**Figure 4:**
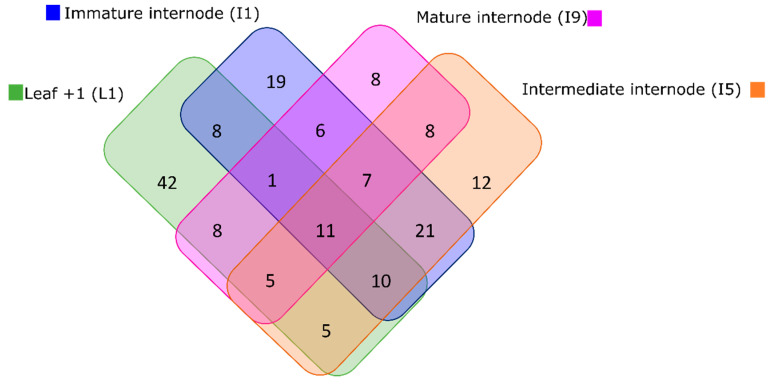
The Venn diagram shows that Leaf +1 presented a higher number of identified metabolites in the metabolite profiles generated via HPLC-MS of each tissue collected throughout sugarcane SP80-3280 development in both fields.

The highest number of metabolites (103 XCMS features matching to 90 unique KEGG metabolites) was detected in L1, the source, decreasing down the stem towards I9, the sink (74 XCMS features matching to 54 unique KEGG metabolites). In L1 most of the metabolites suggested by ProbMetab were tissue-specific (42 unique KEGG metabolites) and merely 11 metabolites were shared among all tissues (Figure 4). Tissue-specific metabolites were identified in all four tissues analyzed and the number decreased from the source to the sink tissue (Figure 4).

**Figure 5:**
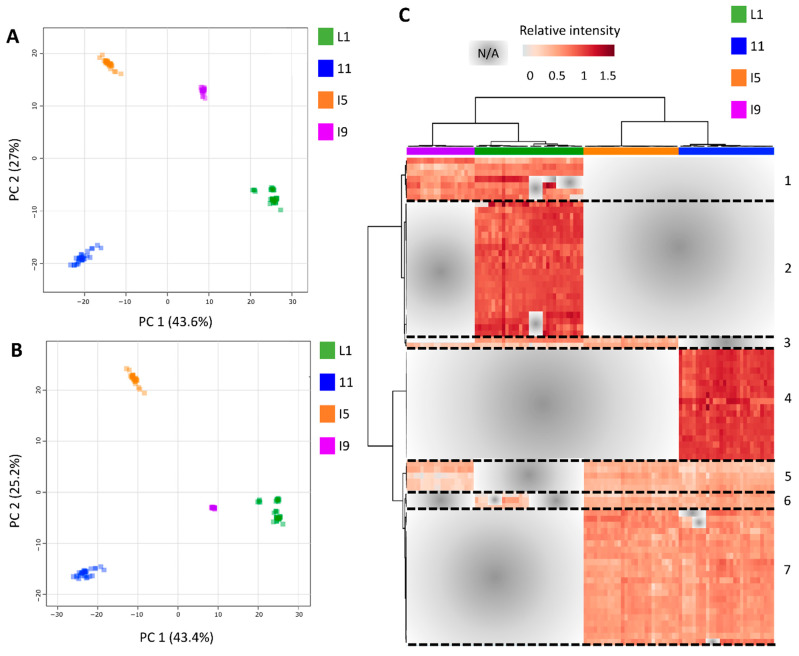
Discriminant models of the metabolomics data demonstrated that most of the variation within metabolomics profiles is attributed to the differences between the distinct anatomical tissues. (**A**) PCA and (**B**) PLS−DA models generated from dry weight (DW) normalized and log10 transformed metabolomics data, combining all tissues (L1, I1, I5, and I9), sampling points (C1, C2, C3, and C4) and experimental fields (F1 and F2) (component 1 Q2 = 0.98761, R2 = 0.9882 and component 2 Q2 = 0.99452, R2 = 0.99516). (**C**) Heatmap representation of the 79 main metabolites responsible for the separations (VIP scores ≥1.0 from PLS-DA analysis). The list of the 79 metabolites names is available in the Table S16.

When the processed metabolomics datasets from all tissues (L1, I1, I5, and I9), sampling points (C1, C2, C3, and C4) and fields (F1 and F2) were concatenated for the generation of PCA and PLS-DA models, clear separations were observed between the four tissues (Figure 5) proving that the majority of the metabolic variation can be explained regarding the differences between the different tissues (L1, I1, I5 and I9) as was observed in the transcriptomics data (Figure 3).

Following the heatmap analysis, five main clusters (and two minor ones) were observed, separating the different tissues (Figure 5C, Table S16). Cluster one consisted of 7 highly abundant tissue specific metabolites for the L1 and I9, mainly glucosinolate biosynthesis (2-(2′-Methylthio)ethylmalic acid), tyrosine precursors, phenylpropanoids, and flavonoids). Cluster two consisted of 22 tissue specific metabolites for L1, mainly containing nucleic acids and nucleotides, such as uracil, 5-Thymidylic acid, deoxyuridine monophosphate (dUMP), and Deoxyinosine monophosphate (dIMP). Additionally, a key component from the Calvin cycle and glycolysis, 3-Phosphoglycerate (3PG) was abundant in the L1 tissue. Cluster three consisted of 18 metabolites exclusively detected in the I1 tissue, mostly represented by alkaloids, such as secologanin, 4-Hydroxyphenylacetaldehyde, Hypoxanthine and 7-Methyluric acid. Purine and Zeatin precursors were also abundant in this tissue, as well as the organic osmolyte glycine betaine. Cluster four was made up of 8 compounds tissue specific for the internodes (I1, I5 and I9). These compounds were mainly flavonoids and glucosinolates, such as Kaempferol-3-O-glucoside, naringenin, cyanidin 3-glucoside, S-(4-Methylthiobutylthiohydroximoyl)-L-cysteine, and Indolylmethyl- desulfoglucosinolate. Lastly, cluster five consisted of 22 metabolites present in high abundance in the I1 and I5 tissue. This cluster was represented by a wider variety of metabolites including amino acid and amino acid intermediates, sugars and nucleic acids from primary metabolism, and flavonoids and phenylpropanoids from secondary metabolism, such as ethylene precursors, L-proline, spermidine, p-coumaric acid, ferulic acid.

### 3.4. Multi-Omics Integration Highlighted Three Main Metabolic Categories in All Four Tissues: Amino Acid Metabolism, Biosynthesis of Secondary Metabolites, and Xenobiotics Biodegradation and Metabolism

The main purpose of this study was to evaluate the transcriptomics and metabolomics of sugarcane during development until ripening in two different seasons. As we have demonstrated, the main source of variation in both fields are the different sugarcane tissues therefore, we combined both metabolomics and transcriptomics using the multi-omics factor analysis (MOFA) [33] for the tissues and fields separately (Figures S4–S9). To discover the principal sources of variation in metabolic pathways (and not in the metabolites individually), we first estimated metabolic pathway activities from the metabolite profiles using the PAPi algorithm [30] to use as an input for MOFA. As metabolomics and transcriptomics data modalities have different dimensionalities, and bigger data modalities tend to be overrepresented [33], we filtered the transcriptomics data by selecting the genes coding for the enzymes in the metabolic pathways that were identified using the PAPi tool.

Based on the combination between metabolomics and transcriptomics, it was possible to point out the main metabolic pathways containing both the identified metabolites and the transcripts of the genes coding for the enzymes directly upstream or downstream of these specific metabolites that are responsible for the principal sources of variation.

#### 3.4.1. Leaf +1 (L1)

For the L1 tissue, it was found that the main metabolic pathways, based on the KEGG BRITE classification (http://www.genome.jp/kegg/, accessed on 17 December 2019), that were activated and altered throughout development were amino acid metabolism (36%), biosynthesis of secondary metabolites (29%), xenobiotics biodegradation and metabolism (21%), carbohydrate metabolism (7%), and metabolism of cofactors and vitamins (7%). Most of the GO terms generated from the transcriptomics data from the MOFA model for the L1 tissue, pointed towards amino acid metabolism and thus complementing the findings reported from the metabolomics data, as shown in Table 1. Moreover, the metabolomics data added one crucial extra layer of information since it was able to pinpoint exactly which amino acid and small molecule metabolic pathways are altered in the L1 tissue (and also in the other tissues as described below) throughout development, namely phenylalanine, tyrosine and tryptophan (Phe, Tyr, Trp) metabolism, and cyanoamino acid metabolism. The secondary metabolic pathways that were altered were phenylpropanoid biosynthesis, flavonoid biosynthesis, glucosinolate biosynthesis, and tropane, piperidine and pyridine alkaloid biosynthesis (Table 1). However, from a transcriptomics point of view, no exact and direct GO terms regarding secondary metabolism were identified. In both metabolomics and transcriptomics data modalities, cofactor metabolism was identified. Yet again, the metabolomics data indicated that it specifically referred to ubiquinone and other terpenoid-quinone biosynthesis under the KEGG BRITE classification “metabolism of cofactors and vitamins”. The only carbohydrate metabolism pathway that was sufficiently activated and altered to be picked up by and included in the MOFA model generated for the L1 tissue, and that also shared corresponding variance with the transcriptomics data, was the pentose phosphate pathway (PPP) as shown in Table 1. The presence of the benzoate degradation, aminobenzoate degradation, and toluene degradation metabolic pathways identified in the metabolomics data modality represented the non-plant pathways from the main KEGG BRITE classification “xenobiotics biodegradation and metabolism”. Interestingly, this phenomenon was also supported by the transcriptomics data in which “drug metabolic process” was one of the GO terms that was identified (Table 1).

#### 3.4.2. Immature Internode (I1)

In I1, 39% of the altered pathways represented secondary metabolite biosynthesis such as isoflavonoid biosynthesis, glucosinolate biosynthesis, flavone and flavonol biosynthesis, phenylpropanoid biosynthesis, isoquinoline alkaloid biosynthesis, and betalain biosynthesis (Table 1, metabolomics data modality), all known to be involved in plant stress response and in the synthesis of lignin precursors. From the 18 GO terms that were highlighted in the MOFA model for I1, 9 indicated that the plants were in fact responding to internal and/or external stressors (Table 1, transcriptomics data modality). Amino acid metabolism represented 16.5% of the altered pathways from the metabolomics data modality, specifically phenylalanine metabolism, tyrosine metabolism, and beta-alanine metabolism. However, no directly linked GO terms were identified regarding these pathways. Yet again, non-plant metabolic pathways representing 28% of the altered pathways were identified in the I1 tissue, including methane metabolism, metabolism of xenobiotics by cytochrome P450, neomycin, kanamycin and gentamicin biosynthesis, monobactam biosynthesis, and biosynthesis of vancomycin group antibiotics. The presence of GO terms from the transcriptomics data modality related to fungal and inorganic substance responses, as well as multi-organism processes support the metabolomics data that indicated that non-plant organisms were in the plants’ proximity, either in their internal or external environment. The remaining metabolic pathways included lipid metabolism and metabolism of cofactors and vitamins, each representing 5.5% of the altered pathways. On the other hand, for the transcriptomics data modality, GO terms related to the cell cycle were identified (Table 1).

#### 3.4.3. Intermediate Internode (I5)

From the activated pathways identified by the MOFA models for I5, all except for one pathway, equally represented the same percentage (10% each) of altered pathways when considering all of the altered pathways. At the top of the list for altered pathways with regards to the metabolomics data, zeatin biosynthesis is listed (Table 1). When analyzing the transcriptomics data, no direct links could be made with regards to zeatin biosynthesis, however the GO term nucleobase-containing small molecule metabolic process was identified (Table 1, transcriptomics data modality), which is most likely due to the identification of the purine metabolism, which is a metabolic pathway that was in fact highlighted in the metabolomics data modality by MOFA. Since adenine is a purine and a zeatin precursor, the possibility exists that these pathways go hand-in-hand and are altered during development in the I5 tissue. The metabolic pathways supporting the GO terms carbohydrate metabolic process and cofactor metabolic process were highlighted as propanoate metabolism and pantothenate and CoA biosynthesis, respectively (Table 1). Tyrosine metabolism was one of the altered amino acid metabolic pathways. Yet again, activated pathways not naturally found in plants were highlighted, representing 30% of the total altered pathways in the I5 tissue. This finding was in fact corroborated by both the metabolomics and transcriptomics data seeing that metabolic pathways such as naphthalene degradation, chlorocyclohexane and chlorobenzene degradation, and styrene degradation were identified, and GO terms related to drug metabolism, detoxification processes, and responses to toxic substances were highlighted from the transcriptomics data (Table 1).

#### 3.4.4. Mature Internode (I9)

Lastly, for I9, amino acid metabolism and carbohydrate metabolism each represented 20% of the altered pathways. Biosynthesis of secondary metabolism and xenobiotics biodegradation and metabolism represented 10% and 30%, respectively. Most of the gene expression data for this tissue pointed towards alterations in aromatic amino acid metabolism, specifically tryptophan metabolism as pinpointed by the metabolomics results (Table 1). The glyoxylate and dicarboxylate metabolism pathway was identified as being altered in I9, which provides intermediates for carbohydrate biosynthesis, most likely for the pentose and glucuronate metabolism pathway since this pathway was also identified in the metabolomics data modality. These observations can be supported by the carbohydrate metabolic process GO term identified in the transcriptomics data modality (Table 1). Non-plant pathways were detected again, in conjunction with the GO term drug metabolic process (Table 1), thus indicating the presence of microorganisms in the plants’ internal and/or external environment, as were the cases for all the other tissues.

### 3.5. The Two Planting Conditions Imposed Differences on Mainly the Leaves and Mature Internodes, and Some Phenylpropanoids Were Detected Only in “One-Year” Sugarcane Leaves

Since the analysis of all tissues in both fields together highlighted the differences among tissues and the aim was to study the differences in the development of sugarcane in two different planting seasons, we evaluated the metabolite profiles in each tissue separately. When each tissue was separately analyzed and PCA and PLS-DA models were generated, they mainly reflected the differences between the two different conditions. The most striking differences were observed in L1 and I9 as was observed in the transcriptomic data (Figure 3).

**Figure 6:**
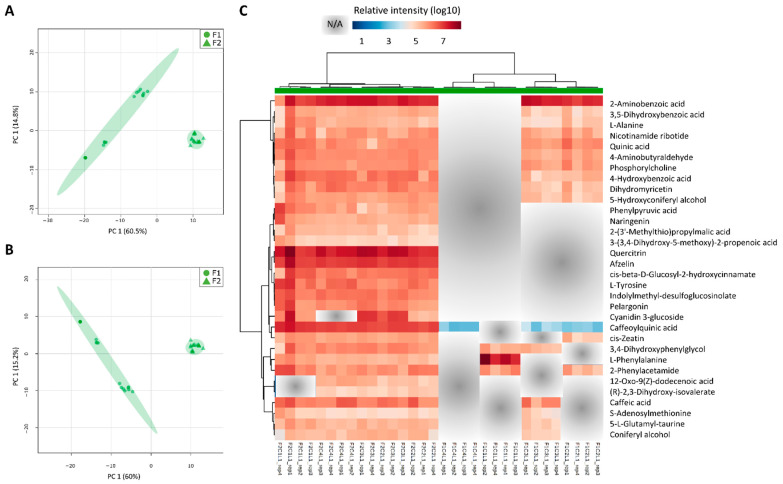
Leaf+1 metabolite profiles are different between fields 1 (F1) and 2 (F2). Discriminant models between field comparisons generated from the normalized and log10 transformed metabolomics data from the leaf +1 (L1) tissues, combining sampling points (C1, C2, C3, and C4) and experimental fields (F1 and F2). (**A**) PCA and (**B**) PLS-DA models for L1 (component 1 Q2 = 0.64317, R2 = 0.70436 and component 2 Q2 = 0.89506, R2 = 0.92523); (**C**) heatmap representation of the 32 main metabolites responsible for the separations (VIP scores ≥1.0 from PLS-DA analysis) and NA refers to non-detected metabolites.

For the L1 tissue, separations were observed with regards to the different planting seasons (F1 and F2) when analyzing the first components (Figure 6A,B).

The metabolites with VIP scores from the PLS-DA models above 1.0 (yielding a total of 32 metabolites), were used for heatmap analysis. An overall view of the heatmap showed that most of the identified metabolites were higher in L1 from F2 as compared to F1 (Figure 6C). These metabolites are mostly involved in plant secondary metabolism and amino acid metabolism: phenylpropanoid biosynthesis, flavone and flavonol biosynthesis, Aminoacyl-tRNA biosynthesis, Phe, Tyr, and Trp metabolism, and 2-Oxocarboxylic acid metabolism. From the list of field specific metabolites in L1 from F2, that means metabolites only detected in the “one-year” sugarcane leaves, flavonoids such as afzelin and quercitrin were abundant (Figure 6). On the other hand, the metabolite cis-Zeatin was absent in all L1 samples from F1 except at C2, 8 months after planting. Caffeoylquinic acid was detected in the L1 from F1 at samplings 2, 3, and 4 (8, 11 and 13 months), however the levels were 2-3 times lower than in the L1 from F2 (Figure 6). As shown in a cluster from the heatmap, caffeic acid was detected in all samples from F2, however it was only detected in L1 from F1 at C3 when there was a decrease in the precipitation (Figure 2A; Table S12). Two metabolites detected only in L1 from plants that reached maturation (C2, C3, and C4 from F2) were (R) 2,3-Dihydroxy-isovalerate (an intermediate in valine, leucine and isoleucine biosynthesis) and 12-oxo-cis-dodec-9-enoic acid (a metabolite of both linolenic and linoleic acids) (Figure 6).

For the I1 tissue, the separations between the two fields were not as prominent as in L1 when the PCA and PLS-DA models were inspected (Figure S10). Nine metabolites were identified based on the PCA and PLS-DA models when considering VIP scores from the PLS-DA models above 1.0. These metabolites corresponded to metabolites involved in primary metabolism, specifically in pentose and glucuronate interconversions and in tyrosine metabolism. The amino acid L-Tyrosine was highly abundant in this tissue, under all conditions. On the other hand, digalacturonic acid D-Galacturonate was more abundant in the I1 from F2 as compared to F1.

For the intermediate internode (I5), even less of a separation could be seen between the fields (F1 and F2) (Figure S11). Nine metabolites were identified with VIP scores above 1.0 from the PLS-DA model and are represented on the heatmap (Figure S11). As for the I1, these metabolites from I5 were yet again from primary metabolism such as amino acid and amino sugar metabolism, and nucleotide metabolism. Two metabolites namely glucosamine and dhurrin were more abundant in the I5 from F1 as compared to F2. On the other hand, stachyose and 5-Methyltetrahydrofolate were higher in I5 from F2 as compared to F1 (Figure S11).

**Figure 7:**
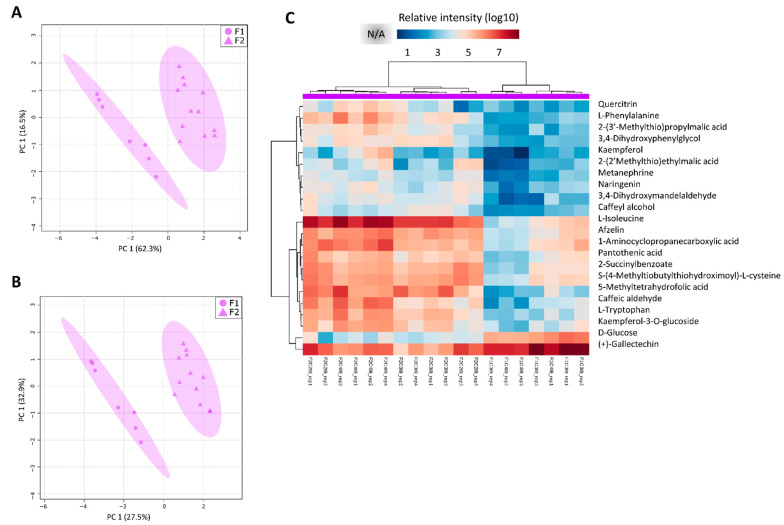
Mature internode metabolite profiles are different between filed 1 (F1) and 2 (F2). Discriminant models for between field comparisons generated from the normalized and log10 transformed metabolomics data from the mature internode (I9) tissues, combining sampling points (C1, C2, C3 and C4) and experimental fields (F1 and F2). (**A**) PCA and (**B**) PLS-DA models for I9 (component 1 Q2 = 0.79453, R2 = 0.84836 and component 2 Q2 = 0.92788, R2 = 0.95558); (**C**) heatmap representation of the 22 main metabolites responsible for the separations (VIP scores ≥ 1.0 from PLS-DA analysis) and NA refers to non-detected metabolites.

Lastly, in correspondence with L1, for the mature internode (I9) greater separations between the different conditions (F1 and F2) were observed (Figure 7). Regarding the metabolites with a VIP score above 1.0 from the PLS-DA model, 22 were identified. All of the metabolites were detected in both fields, but the intensities were found to be different in some samples. These metabolites represent pathways namely biosynthesis of plant secondary metabolism such as flavone and flavonol biosynthesis, phenylpropanoid biosynthesis, flavonoid biosynthesis, biosynthesis of alkaloids, and Phe, Tyr, and Trp biosynthesis. The metabolite (+)-Gallocatechin was abundant in all the samples of the I9 tissue. Most of these metabolites were relatively higher in the I9 from F2 as compared to F1. In contrast, the D-Glucose metabolite, the only sugar out of the 22 metabolites, were higher in the I9 from F1 as compared to F2 (Figure 7).

**Figure 8:**
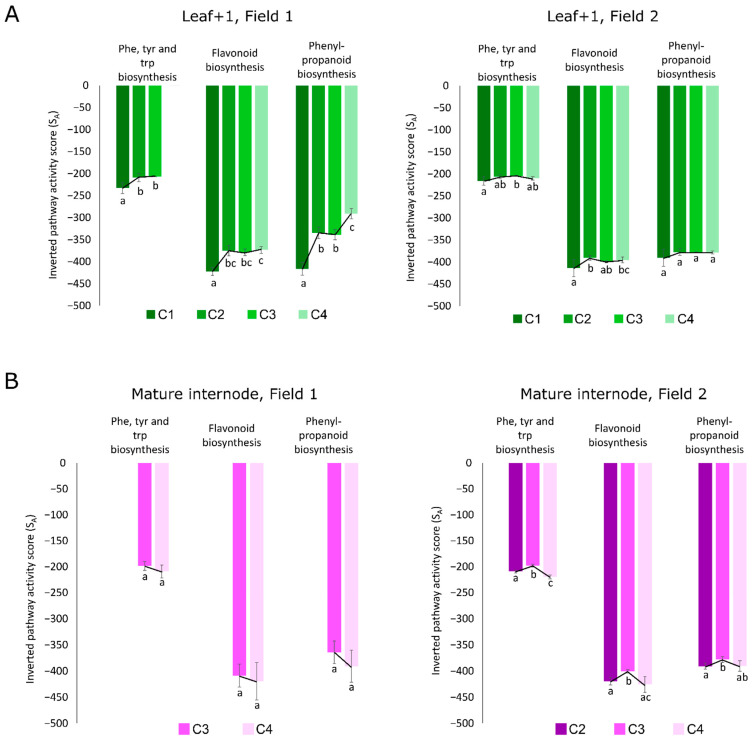
Pathway activities from the phenylalanine, tyrosine and tryptophan biosynthesis, flavonoid biosynthesis and phenylpropanoid biosynthesis. Pathway activities based on the pathway activity profiling (PAPi) tool of (**A**) leaves (L1) from F1 and F2; and of (**B**) mature internodes (I9) from F1 and F2. C1, C2, C3, and C4 refer to sampling points 1, 2, 3 and 4 specifically 4, 8, 11 and 13 months after planting. Bars with different letters (i.e., ‘a’ and ‘b’) indicate statistically significant differences between their activity scores based on ANOVA and Fisher’s LSD (*p* < 0.05) and bars with the same letters (i.e., ‘a’ and ‘a’) do not have activity scores statistically different.

Some of the main metabolites responsible for the separations between F1 and F2 are part of three related metabolic pathways in both L1 and I9: phenylpropanoid biosynthesis (map00940), phenylalanine, tyrosine and tryptophan biosynthesis (map00400), and flavonoid biosynthesis (map00941). The calculated activity scores estimates how these three pathways behave throughout the sugarcane development in both fields in leaves and mature internodes. The higher the score, the lower the activity [30] and thus, to avoid confusion and to facilitate visualization, these scores were inverted for figure representation (Figure 8). In F1 there is an increase in the activity of the three highlighted pathways in leaves throughout development and this was not observed in leaves from F2 (Figure 8A). In contrast, alterations in the activity scores were observed throughout development only in I9 from F2 (Figure 8B). For the L1 from F1, which experienced a period of lower rainfall during the initial vegetative growth phase, and presented no culm formation at C1, the pathway activities of the Phe, Tyr, and Trp biosynthesis, flavonoid biosynthesis, and phenylpropanoid biosynthesis were significantly lower (based on ANOVA and Fisher’s LSD, *p *< 0.05) as compared to when thick culm tissue had been formed at C2, and the vegetative growth and elongation phases were being carried out at C3 and C4 from F1 (Figure 8A). The phenylpropanoid and flavonoid biosynthetic pathways were significantly higher at C4 as compared to C1, however, the Phe, Tyr, and Trp biosynthesis pathway was not even detected (Figure 8A), thus indicating that the flux through this pathway was very low or that it was suppressed at C4. Regarding the pathway activity analysis of the I9 in F1 of the plants in the vegetative growth and elongation phase (C3 and C4), no significant (based on Student’s *t*-test, *p*-value < 0.05) alterations were observed (Figure 8B). On the other hand, in F2, the pathway activities of the Phe, Tyr, and Trp biosynthesis, flavonoid biosynthesis, and phenylpropanoid biosynthesis were significantly higher in the I9 at C3 when the plants experienced water deficit (Figure 8B).

The metabolic intermediates in these pathways and the SAS (EST contigs) directly related to the metabolites (via the specific enzymes directly upstream or downstream of these metabolites on the KEGG database) were identified (Table S17) and the expression level was clustered in heatmaps (Figures S12–S14). A higher number of natural antisense transcripts (NATs) was found to be present in clusters with expression profile differences between F1 and F2 in Phe, Tyr, and Trp biosynthesis (Figure S12) and phenylpropanoid biosynthesis (Figure S13). The overall expression of NATs was absent in the L1 tissue from F1 and present in F2 (13 NAT in Phe, Tyr, and Trp biosynthesis and 17 NATs in phenylpropanoid biosynthesis), except for the NAT of shikimate kinase (SCCCST2004A03.g), 3-phosphoshikimate 1-carboxyvinyltransferase (SCCCLB1003B08.g) (Figure S12) and chalcone synthase (SCVPLR2027D02.g) (Figure S14). In the flavonoid biosynthesis pathway, the NAT of the flavanone 3-dioxygenase (F3H-SCEZLR1009E06.g) was not expressed in the L1 of F1 and was expressed in the L1 of F2 at C3 and C4. The sense transcript of this gene was expressed in all conditions in both F1 and F2 (Figure S14). Some cases of the absence of NAT expression in F1 and the presence of NAT expression in F2 were also observed in the mature internode, however in lower levels (Figures S11–S13).

The two significantly expressed PAL sense (SS) transcripts (SCEQRT1024E12.g and SCCCLR1048D07.g) were expressed in F1 and F2 in both leaves and internodes. In the mature internode both PAL transcripts showed higher expression while in leaf +1 only the SCEQRT1024E12.g transcript was included in the cluster with higher expression (Figure S13). NAT from PAL genes (SCCCLR2002C08.g and SCCCLR1048D07.g) were expressed in L1 from F2 and not in L1 from F2 (Figure S13).

## 4. Discussion

### 4.1. Low Precipitation and Low Temperatures, Two Factors Known to Affect Sugarcane Development, Directs Carbon Flow towards Culm Thickening Instead of Culm Elongation Affecting Ripening

Sugarcane development is mainly affected by four abiotic factors namely water availability [40], temperature variability [41], soil properties [42], and solar radiation [43]. In this study, alterations regarding precipitation and temperature led to different developmental patterns of SP80-3280 planted in two seasons of the year. The different developmental patterns were established during the early growth phases, around four months after planting, producing “one-and-a-half-year” and “one-year” sugarcane as described by Medeiros Barbosa (2015) [44]. For the “one-and-a-half-year” sugarcane from the F1, the observed retarded vegetative growth during the initial months after planting is attributed to the lower precipitation and lower AGDD since it has been proved that internode development and culm elongation are the most sensitive morphological alterations in sugarcane affected by water deficits [45]. We could calculate the AGDD values for SP80-3280 ripening in both seasons (5861 °C for the onset of maturation in F1 and 4833 °C in F2) which for our knowledge had never been done for this variety.

Thicker culms for the “one-and-a-half-year” sugarcane were also observed, supporting the findings by Cardozo (2015) [46] which stated that when tillering occurs early in development under lower precipitation and temperatures, thicker culms are observed. This suggests that under conditions with lower amounts of available water, the carbon flow is directed towards culm thickening and lignification instead of culm elongation. This speculation is supported by other relative studies where a decrease in water availability resulted in the activation of stress response genes that overlapped with genes involved in culm lignification processes [17,47].

It is known that sugarcane has the ability to tolerate early phase water deficits without significantly affecting future yields [48], as was the case with “one-and-a-half-year” sugarcane plants seeing that the vegetative growth and elongation phases were initiated as soon as temperature and precipitation increased. However, when lower precipitation and lower temperatures are experienced during the rapid culm elongation phase, as was the case for the “one-year” sugarcane plants (F2), the ripening phase is initiated due to the reduction in sink strength for structural growth [49], and in this case, yield loss can be substantial [50]. Medeiros Barbosa (2015) [44] reported that “one-and-a-half-year” sugarcane has higher yields (>120 t/ha) as compared to “one-year” sugarcane (<100 t/ha).

### 4.2. Molecular Profiles of Sugarcane Tissues Reflected Their Roles in the Plant and the Higher Number of Metabolites Identified Indicates an Improved Method for Conducting Sugarcane Metabolomics Studies

The majority of the variation within the transcriptomics and metabolome datasets could be explained by the differences between the four tissues. From a biotechnological perspective, these types of genes with tissue-specific expression can possibly be considered as strong candidates for providing promoters that can be used for a more controlled expression of transgenes in crops [51].

Genes involved in secondary metabolic processes and organic acid metabolism were highly expressed in the leaves, in agreement with the results from metabolomics data analysis in which most of the metabolites responsible for the separation of the different anatomical tissues formed part of secondary metabolism and organic acid metabolism as well. Genes responsible for specialized carbohydrate metabolism such as cell wall organization and construction, and sugar metabolism were highly expressed in all internodes. It is well known that cell wall construction and lignification increase down the sugarcane culm [52].

In plants, the leaf tissue is one of the main elements responsible for perceiving and responding to changes in the external environments. Plant secondary metabolism was evolved to allow plants to perceive and adapt to these changing environments [53], thus justifying the high expression of genes and the large amounts of secondary metabolites in the leaf tissue reported here. Additionally, the leaf is also responsible for supporting the growth of the apical meristem, or the immature internode in this case, which is actively growing and elongating. These tissues require building blocks such as amino and organic acids from the source tissue as reported here, thus justifying the high expression of these genes involved in organic amino acid biosynthesis. A cluster of transcripts that were highly expressed in the immature and intermediate internodes contains processes responsible for cell duplication and growth, and culm elongation, thus supporting the role of these tissues as actively growing tissues. Looking specifically at metabolites themselves and not the metabolic pathways in which they participate, L-proline, presented high abundances in the immature and intermediate internodal culm tissue. These results are in agreement with a previous study that reported this amino acid as one of the major free amino acids in sugarcane culm tissue [54]. It has also been shown that this amino acid is present in low amounts in the mature sugarcane culm tissue [55], as was found in our study since the abundance decreased down the culm. Since the immature and intermediate internodes are actively growing, and culm elongation is occurring in these regions, higher levels of proline may be required since this metabolite has been described as a key player needed for cell elongation in previous studies [56,57]. Lastly, a cluster of transcripts related to the upstream and downstream processes of lignification were highly expressed in the more mature sugarcane tissues, thus corresponding to previous studies conducted on the lignification of the sugarcane culm [52,58].

The highest number of metabolites were present in the leaves and decreased down the stem (Figure 4) which agree with previous metabolomics studies conducted on sugarcane tissue [59]. Interestingly, a relatively higher number of metabolites were reported in the current study in comparison to other untargeted sugarcane metabolomics studies in the literature [59,60,61], thus, contributing to knowledge gaps regarding the sugarcane metabolome and presenting an improved method for conducting sugarcane metabolomics studies.

### 4.3. Economically Valuable Compounds That Affect Plant Growth and Productivity; Inhibits SARS-CoV-2 and Are Intermediates of Glucosinolates Were Identified

Compounds involved in plant growth that could, in the future, be used to improve sugarcane were identified. Spermidine, a polyamine (PA), was found in higher levels in the immature and intermediate internodes as compared to the leaves and mature internodes (Table S16) and high PA levels have been associated with plant growth and development [62], possibly supporting the high spermidine levels in these specific sugarcane tissues. This is in agreement with the higher expressions of genes with the GO terms related to cell cycle, including chromosome organization and DNA replication (Table 1) detected in the immature and intermediate internodes.

Our results indicated higher levels of p-coumaric acid in the same tissues as spermidine and it has been shown that PAs can bind with phenolic compounds such as hydroxy cinnamic acid, coumaric acid, caffeic acid, or ferulic acid to form CC-PAs [63,64], thus possibly supporting our observations and the positive association between spermidine and p-coumaric acid. Thus, there is increasing evidence that targeting the increase of PAs such as spermidine can positively affect plant growth and productivity [62]. The presence of PA biosynthesis transcripts was also detected in the transcriptomics data retrieved in the present study (Table S4), thus the targeting of these genes will be possible for hypothesis testing in SP80-3280. On the other hand, the metabolite p-coumaric acid by itself has also been linked to plant growth. The exogenous application of p-coumaric acid to chia (*Salvia hispanica*) led to increased shoot elongation and biomass accumulation and presented positive correlations with proline contents [65], as was observed in our case.

Metabolites involved in tyrosine metabolism and glucosinolate biosynthesis were found to be higher in the leaves (L1) and mature internodes (I9) as compared to the intermediate internodes (I1 and I5) studied here (Figure 5). Tyrosine-derived metabolites can act in plant defense, attraction of pollinators, serve in electron transport and form part of the structural support in the cell walls of plants [66] and the production of this derived compounds may be explored via synthetic biology platforms [66]. A metabolite called 2-(2′-Methylthio)ethylmalic acid from the glucosinolate biosynthetic pathway, was also highlighted in the leaves and mature internode (Figure 5, Table S16) It has been shown that glucosinolates have herbivore deterrent [67], fungicidal [68], bactericidal [69], nematocidal [70], and allelopathic properties in plants [71]. In the industry, these glucosinolates from plants have also been greatly explored and exploited for their roles in cancer treatment and the regulation of blood glucose levels in humans [72,73], as biopesticides in crop plants [74] and flavor compounds [71]. It has been described that glucosinolate distribution in plants vary under different environmental conditions [75] and depending on the different plant organs and tissues [71], as was the case here seeing they were higher in the leaves and mature internodes.

The flavonoid quercitrin was detected mainly in sugarcane leaves from F2 (Figure 6). Quercitrin has been recently described as promising inhibitor of SARS-CoV-2 in different studies as follows: quercitrin is a promising inhibitor for protein ADP ribose phosphatase, a receptor protein of SARS-CoV-2; for 3Cl protease, the main protease of SARS-CoV-2 and has prospective binding affinities for papain-like protease (PLpro), all three essential for the virus replication [76,77,78]. Quercitrin also binds and probably inhibits the host cellular serine protease TMPRSS2, that would inhibit the SAR-CoV-2 viral entry into the human host cells [79].

The metabolites above mentioned and the lack of studies in sugarcane metabolomics, highlights the new possibilities for the exploration of the sugarcane metabolome and its use in the production of economically valuable products.

### 4.4. Integration of Transcriptomics and Metabolomics Revealed Alterations in Metabolic Pathways Related to Development and Abiotic Stress in Plants

In most “multi-omic” or “double-omic” studies, the statistical analyses of metabolomics and transcriptomics data was done separately without using a specific tool for their direct integration [80,81,82,83,84], since this type of statistical integration is an extremely complex task. Here, a bioinformatics tool called multi-omics factor analysis (MOFA) [33] was implemented in order to deal with this expected problem regarding the huge, heterogeneous datasets. Specific latent factors (LFs) are then identified which capture the major shared sources of variation across the different data modalities, and shared patterns can be highlighted using this tool. Here, the LFs represented metabolic pathways and their activities, and specific transcripts directly upstream or downstream linked to the metabolites within the highlighted metabolic pathways. The MOFA tool is publicly available in our sugarcane database to integrate data on sugarcane transcriptomics and metabolomics (https://sucest-fun.org/wsapp/, accessed on 30 April 3021).

For the different sugarcane tissues of SP80-3280 analyzed here, the main metabolic systems that were altered throughout development and in response to changes in the external environments were amino acid metabolism, mostly Phe, Tyr, Trp metabolism; and secondary metabolism, mostly phenylpropanoid metabolism and the metabolism of its upstream precursors and/or downstream products. Previous studies on the effects of abiotic stress on plants such sugarcane and Arabidopsis have reported alterations in amino acid metabolism [85,86]. In sugarcane, during drought stress the carbohydrate metabolism is coordinated with the degradation of amino acids probably to provide carbon skeletons for the tricarboxylic acid cycle maybe facilitating recovery after the stress [85]. Aromatic amino acids such as Phe, Tyr, Trp, are the main amino acids being synthesized under stress conditions [86,87] and these aromatic amino acids serve as precursors for secondary metabolites, such as glucosinolates, alkaloids, and phenylpropanoids, which play important roles in plant development [88,89]. Our results from MOFA pointed out these exact mentioned pathways (glucosinolate biosynthesis; tropane, piperidine and pyridine alkaloid biosynthesis; and phenylpropanoid biosynthesis). It is well-known that a period of natural water deficit is necessary for the termination of the vegetative growth phase of sugarcane and the initiation of the maturation phase where sucrose is accumulated in the culms [43,49]. Accordingly, sugarcane plants from our study do in fact experience a period of low rainfall before the maturation observed in F1 after 13 months and in F2 after 11 months. Additionally, GO terms pointing towards abiotic stress response were also highlighted in the MOFA models, showing that stress response genes are altered throughout development in response to changes in the external environment.

Phenylpropanoids, such as flavonoids, are key players in the synthesis of cell wall precursors and function in plant development [90]. Phenylpropanoid pathway and Phenylalanine and Tyrosine biosynthesis are two important pathways with genes differentially expressed during sugarcane stem maturation in high and low fiber genotypes [91]. Since the main phenotypic differences of the plants from the two different fields from our study were related to plant height and culm diameter, our findings regarding this pathway activities indicate a possible role for the phenylpropanoid biosynthetic pathway and its derivative pathway, flavonoid biosynthesis, in sugarcane development and adaptation to changes in the plant’s internal and/or external environments.

### 4.5. Leaves from “One-Year” Sugarcane Present Phenylpropanoids Not Detected in “One-and-a-Half-Year” Sugarcane That May Be Related to the Drying Off and Maturation Detected Early in F2

In order to identify the exact metabolites responsible for the separations between the “one-and-a-half-year” sugarcane from F1 and “one-year” sugarcane from F2 in each tissue, the PCA and PLS-DA models were inspected separately, and the main metabolites responsible for the separations in each tissue (L1, I1, I5 and I9) were highlighted.

Looking at the leaf tissue, the majority of the identified metabolites were in the “one-year” sugarcane plants from F2, which presented earlier maturation at 11 months old as Brix%, Pol % juice, Pol % cane and purity% were higher than 18%, 15.3%, 13% and 85%, respectively [38,39]. After eight months of planting, these plants experienced a period of lower precipitation which led to elongation suppression and the initiation of the maturation phase. At the end of the experiment, an unwanted shift to the reproductive stage (flowering) occurred with a reduction in Brix content.

Out of the 32 metabolites identified in the leaves for being responsible for the separation between the two fields, the majority of them were phenylpropanoids and intermediates of the phenylpropanoid biosynthesis pathway (Figure 6C). It is known that the lack of water affects plant flavonoid biosynthesis. A previous study showed that oxidative and drought stress conditions increased the flavonoid contents in Arabidopsis leaves, specifically anthocyanins [92], as was the case here seeing that pelargonin, which is involved in anthocyanin biosynthesis, was abundant in the leaves of the “one-year” sugarcane plants (Figure 6C).

Afzelin, together with quercitrin above mentioned, are two flavonoids reported to be present in many plant leaves [93,94,95] and were mainly detected in the leaves of the “one-year” sugarcane from F2 (Figure 6C). Afzelin is a flavonol glycoside which is well known for its function as a reactive oxygen species (ROS) scavenger [96].

In addition to the phenylpropanoids that were abundant in the leaves of the “one-year” sugarcane, the monolignols cis-beta-D-Glucosyl-2-hydroxycinnamate, caffeic acid and caffeoylquinic acid, were also abundant (Figure 6C). Monolignols are the monomeric units that form the base of lignin in plants. The phenomenon that these compounds are being produced in non-structural tissues such as leaves has been reported [97], and the expression of monolignol biosynthetic genes are not necessarily correlated with the presence of lignin [98] seeing that they can be used to produce a wide range of other phenylpropanoid derived compounds [99,100]. The study of transporters of monomers could also provide useful insights on how these compounds are synthesized in the leaves and transported to the tissues that provide structural support to the plants, such as the culm.

In the immature and intermediate culm tissue, the main metabolites highlighting the relatively small differences between the two fields (F1 and F2), that is between “one-year” and “one-and-a-half-year” sugarcane, were found to be mostly involved in primary metabolism (Figures S9 and S10). However, two metabolites namely digalacturonic acid and d-galacturonate, which are pectic cell wall monomers, were higher in the immature internodes of the “one-year” sugarcane plants from F2, which presented thicker culms and were shorter (Figure 2). These compounds are considered important constituents of plant biomass and their microbial degradation has been investigated for converting pectin-rich agricultural residues, such as sugar beet pulp, citrus waste and apple pomace, into value added products [101]. At this moment, these types of residues are sold as animal feed at low values [101]. Since the immature and intermediate internodes of sugarcane are not rich in sugar, the possibility exists of exploring these aerial parts for value added products when thick culms are present and as most of the pectin is easily extracted from the sugarcane cell walls [102].

The cyanogenic glucoside, dhurrin, synthesized from tyrosine, was initially identified in sorghum (Sorghum bicolor) and it is reported that this compound is abundant in intermediate plants and absent in mature plants [103]. A study was done to determine if the dhurrin biosynthetic genes are present in sugarcane by data mining through the SUCEST-FUN database. The authors reported sugarcane enzymes with high similarity to those of sorghum, suggesting that this pathway might be conserved in these organisms [104]. The results from our study confirmed the presence of dhurrin in sugarcane and it was present in higher amounts in intermediate internodes of one-and-a-half-year sugarcane (young and mature plants) which presented delayed maturation.

The oligoarray used in this study for transcriptomics studies was already used for antisense transcription detection [36,105,106]. Our identification of NAT expression is in agreement with previous observations from our group of NAT expression in the phenylpropanoid and Phe, Tyr and Trp metabolisms in the ancestral genotypes (*S. officinarum*, *S. spontaneum* and *S. robustum*) and a commercial hybrid (RB867515). To deepen our study on the high number of identified NATs (49 in L1 and I9 from the three highlighted pathways), RNAseq approaches must be used to characterize the size and sequences of the NATs that where here identified as the Agilent oligoarray only informs us that a sequence of at least the size of the probe (60 mer) is being expressed [36].

As cited earlier, some of the main metabolites responsible for discriminating between F1 and F2 are part of three metabolic pathways in both leaf 1 and mature internode: Phenylpropanoid biosynthesis, Phe, Tyr, Trp biosynthesis and Flavonoid biosynthesis. The developmental profile of sugarcane is established within the initial months after planting, and long-term adjustments to plant growth and culm thickening take place. The thicker culm phenomenon of the “one-and-a-half-year” sugarcane plants was the first indication that secondary metabolism is affected when there is a shortage of water within the initial months after planting. Studies on maize [107], wheat [108] and rice [109] also reported thicker culms when the plants were subjected to water limitations. These reports justify the selection of the pathways (Phe, Tyr, Trp biosynthesis, phenylpropanoid biosynthesis and flavonoid biosynthesis) for further inspection seeing that they are the main pathways implicated in culm formation, culm metabolism, and environmental response. Phe, Tyr, Trp are the main aromatic amino acids for the synthesis of various plant secondary metabolites [110], and phenylpropanoid biosynthesis, the former flowing into the latter and thus leading to the production of lignin precursors. Intermediates of the phenylpropanoid biosynthetic pathway can be directed towards flavonoid biosynthesis, producing secondary metabolites that serve a multitude of functions including an antioxidant role [111], and conferring drought tolerance in some plant species [92,112].

## 5. Conclusions

The main goal of this project was to integrate transcriptomics and metabolomics data to study sugarcane development in two different seasons. Morphological and technological data evidenced that precipitation and temperature have significant influences on sugarcane development, and that the developmental profiles are established within the first four months of planting.

For the “one-and-a-half-year” sugarcane from the first field experiment, the observed retarded vegetative growth could be attributed to the lower precipitation and lower temperatures since it has been proved that internode development and culm elongation are the most sensitive morphological alterations in sugarcane affected by water deficits. It was also concluded that SP80-3280 can support early phase water limitation by continuing photosynthate production and carbon assimilation, however the carbon flow is directed towards culm thickening instead of culm elongation or sugar accumulation. This is known as a long-term adjustment to the changes in the external environment. By planting SP80-3280 under low precipitation and temperatures, more biomass could be produced in the form of fiber over a longer period of time, thus highlighting optimal conditions for the cultivation of SP80-3280 when fiber accumulation is desired. In contrast, when higher sucrose accumulation is desired, rapid early-stage culm development and elongation are necessary, followed by a period of moderate water deficit in order to shift from the vegetative growth to the ripening phase.

With the untargeted metabolomics pipeline, we identified 90, 84, 79, and 54 metabolites in the leaf +1, immature internodes, intermediate internodes and mature internodes of SP80-3280, which were assigned to 67, 70, 69, and 64 activated KEGG metabolic pathways. The high number and the chemical diversity of the metabolites detected here contributed to knowledge gaps regarding the relatively unexplored sugarcane.

Economically valuable compounds in specific sugarcane tissues were identified, specifically secondary metabolism compounds such as phenolic compounds and glucosinolates, which were higher in the leaves and the mature culm tissue. Compounds with economic value for having biopesticide, allelopathic, antioxidant, pharmaceutical, flavor enhancing and antiviral properties were also identified. In the sugar and ethanol production industries, the leaves and remaining bagasse after the sucrose extraction processes are not currently being used to their full potential, thus the metabolites highlighted here and their economic importance might aid in the decision to explore the opportunities of the improved utilization of these tissues. One possibility would be to increase production in the specific tissue by producing sugarcane under different conditions, by controlling the outcome of the developmental profiles as was seen in this study, or by engineering pathways.

Overlaps between the metabolomics and transcriptomics data were observed when they were analyzed separately via data dimensionality-reduction techniques, as well as when they were directly integrated in an unsupervised manner using the MOFA tool. The combination of metabolomics and transcriptomics data highlighted the metabolites and genes from amino acid metabolism, secondary metabolism, and the metabolism of its upstream precursors and/or downstream products and helps to determine and to validate if a biosynthesis pathway is present in an organism.

**Figure 9:**
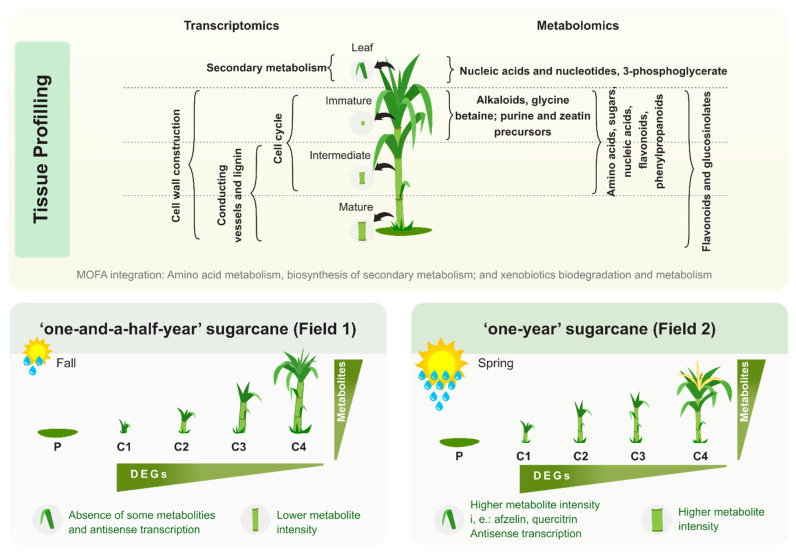
Two plantings of SP80-3280 were carried out in two seasons of the year with different climatic conditions that resulted in the “one-and-a-half-year sugarcane” (Field 1) and “one-year sugarcane” (Field 2) with differences in the plant width, height, brix content and ripening time. A systems biology approach was taken to study sugarcane development including physiological, morphological, agrotechnological, transcriptomics, and metabolomics analyses in plants with 4 (C1), 8 (C2), 11 (C3), and 13 (C4)-months after planting (P) in the leaf +1, immature, intermediate and mature internodes. The number of differentially expressed genes (DEGs) decreased during plant development (from C1 to C4) and the number of metabolites identified decreased from L1 to I9. Most of the variation found within the transcriptomics and metabolomics profiles is attributed to the differences among the distinct anatomical tissues and a summary of the functional categories identified in the tissue profiling is shown here. The integration of both omics highlighted three main metabolic categories as the principal sources of variation in all tissues. The leaves and mature internodes from the two fields presented different intensities of metabolites and natural antisense transcripts with some of them only detected in the leaves of “one-year” sugarcane.

For the different tissues of SP80-3280 analyzed, the differences in metabolite profiles between F1 and F2 were found in the leaf and mature internodes with metabolites and genes from the Phe, Tyr, Trp biosynthesis pathway, the phenylpropanoid biosynthesis pathway and the flavonoid pathway. Leaves from “one-year” sugarcane present phenylpropanoids and antisense transcript expression of many genes not detected in “one-and-a-half-year” sugarcane. Here we present a summary figure of the main results (Figure 9).

Since the main phenotypic differences of the plants from the two different fields from our study were related to plant height and culm diameter, the reported findings regarding the pathway activities conclude that the phenylpropanoid biosynthetic pathway and its derivative pathway, flavonoid biosynthesis, are crucial to plant development and adaptation to changes in the plant’s internal and/or external environments. It is suspected that the Phe, Tyr, Trp biosynthesis pathway is a key pathway which will provide energy and intermediates for the proper functioning of the above-mentioned pathways.

## Figures and Tables

**Table 1:** An integrative view of the combined output results from MOFA for the leaf +1 (L1), immature (I1), intermediate (I5), and mature internodal (I9) tissues.

Tissue	Transcriptomics Data Modality		Metabolomics Data Modality	
	GO ID	GO Term	KEGG Map ID	KEGG Map Name
L1	GO:0044281	small molecule metabolic process	map00940	Phenylpropanoid biosynthesis
	GO:0006520	cellular amino acid metabolic process	map00941	Flavonoid biosynthesis
	GO:0043436	oxoacid metabolic process	map00360	Phenylalanine metabolism
	GO:0006082	organic acid metabolic process	map00640	Cyanoamino acid metabolism
	GO:0019752	carboxylic acid metabolic process	map00966	Glucosinolate biosynthesis
	GO:1901566	organonitrogen compound biosynthetic process	map00030	Pentose phosphate pathway
	GO:0008652	cellular amino acid biosynthetic process	map00400	Phenylalanine, tyrosine and tryptophan metabolism
	GO:1901605	alpha-amino acid metabolic process	map00380	Tryptophan metabolism
	GO:0044283	small molecule biosynthetic process	map00362	Benzoate degradation
	GO:1901607	alpha-amino acid biosynthetic process	map00960	Tropane, piperidine and pyridine alkaloid biosynthesis
	GO:0016311	Dephosphorylation	map00350	Tyrosine metabolism
	GO:0051186	cofactor metabolic process	map00623	Toluene degradation
	GO:0017144	drug metabolic process	map00627	Aminobenzoate degradation
	GO:0055086	nucleobase-containing small molecule metabolism	map00130	Ubiquinone and other terpenoid-quinone biosynthesis
I1	GO:0010035	response to inorganic substance	map00943	Isoflavonoid biosynthesis
	GO:0010077	maintenance of inflorescence meristem identity	map00966	Glucosinolate biosynthesis
	GO:0009414	response to water deprivation	map00944	Flavone and flavonol biosynthesis
	GO:0006952	defense response	map00680	Methane metabolism
	GO:0009415	response to water	map00360	Phenylalanine metabolism
	GO:0005980	glycogen catabolic process	map00940	Phenylpropanoid biosynthesis
	GO:0046398	UDP-glucuronate metabolic process	map00998	Biosynthesis of secondary metabolites-unclassified
	GO:0050832	defense response to fungus	map00130	Ubiquinone and other terpenoid-quinone biosynthesis
	GO:0006950	response to stress	map00980	Metabolism of xenobiotics by cytochrome P450
	GO:0045944	positive regulation of transcription by RNA polymerase II	map00524	Neomycin, kanamycin and gentamicin biosynthesis
	GO:0006457	protein folding	map00950	Isoquinoline alkaloid biosynthesis
	GO:0061077	chaperone-mediated protein folding	map00350	Tyrosine metabolism
	GO:0006298	mismatch repair	map00564	Glycerophospholipid metabolism
	GO:0050896	response to stimulus	map00965	Betalain biosynthesis
	GO:0032508	DNA duplex unwinding	map00261	Monobactam biosynthesis
	GO:0051704	multi-organism process	map00410	beta-Alanine metabolism
	GO:0009620	response to fungus	map01055	Biosynthesis of vancomycin group antibiotics
	GO:0032392	DNA geometric change	map00480	Glutathione metabolism
I5	GO:0005975	carbohydrate metabolic process	map00908	Zeatin biosynthesis
	GO:0051186	cofactor metabolic process	map00954	Stilbenoid, diarylheptanoid and gingerol biosynthesis
	GO:0017144	drug metabolic process	map00564	Glycerophospholipid metabolism
	GO:0042737	drug catabolic process	map00230	Purine metabolism
	GO:0098754	Detoxification	map00640	Propanoate metabolism
	GO:0009636	response to toxic substance	map00770	Pantothenate and CoA biosynthesis
	GO:0045229	external encapsulating structure organization	map00626	Naphthalene degradation
	GO:0044281	small molecule metabolic process	map00361	Chlorocyclohexane and chlorobenzene degradation
	GO:0055086	nucleobase-containing small molecule metabolism	map00350	Tyrosine metabolism
	GO:0098869	cellular oxidant detoxification	map00643	Styrene degradation
I9	GO:0006470	protein dephosphorylation	map00860	Porphyrin and chlorophyll metabolism
	GO:0009072	aromatic amino acid family metabolic process	map00630	Glyoxylate and dicarboxylate metabolism
	GO:0016311	dephosphorylation	map00998	Biosynthesis of secondary metabolites-unclassified
	GO:0005975	carbohydrate metabolic process	map00040	Pentose and glucuronate interconversions
	GO:0009073	aromatic amino acid family biosynthetic process	map00680	Methane metabolism
	GO:0016053	organic acid biosynthetic process	map00380	Tryptophan metabolism
	GO:0046394	carboxylic acid biosynthetic process	map00523	Polyketide sugar unit biosynthesis
	GO:0044281	small molecule metabolic process	map00340	Histidine metabolism
	GO:0017144	drug metabolic process	map00261	Monobactam biosynthesis
	GO:0006520	cellular amino acid metabolic process	map00983	Drug metabolism-other enzymes

## Data Availability

Transcriptomics data is available in the https://sucest-fun.org/wsapp/crossexperiment.do (accessed on 3 December 2021) in the Cane Gene Expression sheet (Growth & Maturation Project). The microarray data have been deposited in NCBI’s Gene Expression Omnibus and are accessible through GEO Series accession numbers GSE124990 and GSE125067 Metabolites identified are available in the Supplementary Tables and in the https://sucest-fun.org/wsapp/searchMetabolomics.do (accessed on 3 December 2021) in the Cane Metabolome sheet. Supplementary Material is also available at https://sucest-fun.org/wsapp/publication.do (accessed on 3 December 2021).

## Supplementary Materials

The following are available online at https://www.mdpi.com/article/10.3390/cells10123451/s1. Figure S1. Soil water balance and accumulated growing degree days (AGDD) in field 1 (black bar) and field 2 (grey bar. Figure S2. Instantaneous photosynthetic rate (A), transpiration rate (B), stomatal conductance (C), water use efficiency (D), number of stems per clamp (E) and soluble carbohydrates in leaf (F) in field 1 and field 2 at 4 (C1), 8 (C2), 11 (C3) and 13 (C4)-month-old plants. Figure S3. Sucrose contents in (A) field 1 and in (B) field 2. Figure S4. Study overview and data types for leaf +1 (L1) collected from experimental field 1. Figure S5. Study overview and data types for leaf +1 (L1) collected from experimental field 2. Figure S6. Study overview and data types for immature internode (I1) collected from experimental field 1. Figure S7. Study overview and data types for immature internode (I1) collected from experimental field 2. Figure S8. Study overview and data types for the intermediate internodes (I5) collected from experimental field 1. Figure S9. Study overview and data types for mature internodes (I9) collected from experimental field 2. Figure S10. Discriminant models for the immature internode (I1) between fields. Figure S11. Discriminant models for the intermediate internode (I5) between fields. Figure S12. Natural antisense transcript expression of genes from the Phenylalanine, tyrosine and tryptophan biosynthesis pathways differs between field 1 and field 2 in leaves. Figure S13. Natural antisense transcript expression of genes from the phenylpropanoid biosynthesis pathways differs between field 1 and field 2 in leaves. Figure S14. Gene expression of genes from the flavonoid biosynthesis pathways in field 1 and field 2. Table S1. Scheme of the microarray hybridizations performed and samples used for the transcriptomic analysis. Table S2. Gradient information for the high-performance liquid chromatography coupled mass spectrometry (HPLC-MS) runs using a pentafluorophenyl propyl ligand column (Phenomenex Luna PFP, 100 mm × 4.60 mm × 2.6 μm). Table S3. Technological parameters in sugarcane culms in field 1 and field 2. Table S4. Sugarcane SAS significantly expressed in the CaneRegNet oligoarray. Table S5. The gene ontology (GO) IDs and terms identified in each cluster from the heatmap analysis using the top 500 most variable genes responsible for the variations in the different tissues in the transcriptomic study. Table S6. Differentially expressed genes (DEGs) between two different ages (C1 vs. C2, C2 vs. C3, C3 vs. C4) in field 1 (F1) and field 2 (F2) in the four different tissues, namely leaf +1 (L1), immature internode (I1), intermediate internode (I5) and mature internode (I9). C1, C2, C3 and C4 refer to 4, 8, 11 and 13 months after planting. Table S7. Differentially expressed genes (DEGs) between field 1 and field 2 (F1 vs. F2) in each tissue (L1, I1, I5 or I9). C1, C2, C3 and C4 refer to 4, 8, 11 and 13 months after planting. Table S8. Percentages of the differently transformed metabolomics data generated for the leaf +1 (L1), immature (I1), intermediate (I5) and mature internodal (I9) tissue samples in which the Shapiro Wilk’s test for normality were *p* > 0.05. Table S9. Percentages of the log10 transformed metabolomics data generated for the leaf +1 (L1), immature (I1), intermediate (I5) and mature internodal (I9) tissue samples, in conjunction with auto scaled and pareto scaled metabolomics data, in which the Shapiro–Wilk’s test for normality were *p* > 0.05. Table S10. Principal Component Analysis (PCA) results generated for the log10 transformed metabolomics data of the leaf +1 (L1), immature (I1), intermediate (I5) and mature internodal (I9) tissue samples when no scaling, auto scaling and pareto scaling techniques were applied on the metabolomics data. Table S11. Number of features detected from XCMS in both positive (pos) and negative (neg) ionization modes; the corresponding amount of XCMS features corresponding to at least 1 KEGG ID; the total possible amount of KEGG IDs detected and the unique KEGG IDs detected in each tissue (L1, I1, I5 or I9). Table S 12. Metabolites detected in the leaf +1 (L1) tissues and their relative intensities. Table S13. Metabolites detected in the immature internode (I1) tissues and their relative intensities. Table S14. Metabolites detected in the intermediate internode (I5) tissues and their relative intensities. Table S15. Metabolites detected in the mature internode (I9) tissues and their relative intensities. Table S16. List of 79 metabolites responsible for the separations between the four different anatomical tissues (VIP scores ≥1.0 from PLS-DA analysis). Table S17. Phenylpropanoid biosynthesis; phenylalanine, tyrosine and tryptophan bio-synthesis and flavonoid biosynthesis pathways from the KEGG database, the pathway map ID, the enzymes within the particular pathway and corresponding SAS in the particular pathway. Supplementary Data 1: Supplementary data with mz, rt and raw intensity in positive and negative ionization modes for each sample; and XCMS ID, KEGG name and probability.

## References

[1] Goldemberg J. (2007). Ethanol for a Sustainable Energy Future. Science.

[2] Gupta R.B., Demirbas A. (2010). Gasoline, Diesel, and Ethanol Biofuels from Grasses and Plants.

[3] Lukas E.N. (2015). Green Economy for Sustainable Development and Poverty Eradication. MJSS.

[4] Cortez L.A.B. (2014). Sugarcane Bioethanol—R&D for Productivity and Sustainability.

[5] Mekonnen Z., Amini A. (2019). Observed and Projected Reciprocate Effects of Agriculture and Climate Change: Implications on Ecosystems and Human Livelihoods. Climate Change and Global Warming.

[6] Wagner de Oliveira M., Cláudio Inácio da Silveira L., Bosco de Oliveira A., Henrique Pereira Barbosa M., Gomes Pereira M., Bezerra Albino Oliveira T. (2019). Sugarcane Production Systems in Small Rural Properties. Multifunctionality and Impacts of Organic Agriculture [Working Title].

[7] Cardozo N.P., Sentelhas P.C. (2013). Climatic Effects on Sugarcane Ripening under the Influence of Cultivars and Crop Age. Sci. Agric..

[8] Krzyzaniak Y., Negrel J., Lemaitre-Guillier C., Clément G., Mouille G., Klinguer A., Trouvelot S., Héloir M.-C., Adrian M. (2018). Combined Enzymatic and Metabolic Analysis of Grapevine Cell Responses to Elicitors. Plant Physiol. Biochem..

[9] Matilla M.A. (2018). Metabolic Responses of Plants Upon Different Plant–Pathogen Interactions. Plant Metabolites and Regulation Under Environmental Stress.

[10] Bhatla S.C. (2018). Plant Growth Regulators: An Overview. Plant Physiology, Development and Metabolism.

[11] Gatehouse A.M.R., Christou P., Klee H. (2004). Engineering of Crops for Improved Agronomic Traits. Handbook of Plant Biotechnology.

[12] Csorba T., Questa J.I., Sun Q., Dean C. (2014). Antisense *COOLAIR* Mediates the Coordinated Switching of Chromatin States at *FLC* during Vernalization. Proc. Natl. Acad. Sci. USA.

[13] Caldana C., Fernie A.R., Willmitzer L., Steinhauser D. (2012). Unraveling Retrograde Signaling Pathways: Finding Candidate Signaling Molecules via Metabolomics and Systems Biology Driven Approaches. Front. Plant Sci..

[14] Schrimpe-Rutledge A.C., Codreanu S.G., Sherrod S.D., McLean J.A. (2016). Untargeted Metabolomics Strategies—Challenges and Emerging Directions. J. Am. Soc. Mass Spectrom..

[15] Manechini J.R.V., da Costa J.B., Pereira B.T., Carlini-Garcia L.A., Xavier M.A., de Landell M.G.A., Pinto L.R. (2018). Unraveling the Genetic Structure of Brazilian Commercial Sugarcane Cultivars through Microsatellite Markers. PLoS ONE.

[16] Souza G.M., Van Sluys M.-A., Lembke C.G., Lee H., Margarido G.R.A., Hotta C.T., Gaiarsa J.W., Diniz A.L., de Oliveira M.M., de Ferreira S.S. (2019). Assembly of the 373k Gene Space of the Polyploid Sugarcane Genome Reveals Reservoirs of Functional Diversity in the World’s Leading Biomass Crop. Gigascience.

[17] Papini-Terzi F.S., Rocha F.R., Vêncio R.Z., Felix J.M., Branco D.S., Waclawovsky A.J., Del Bem L.E., Lembke C.G., Costa M.D., Nishiyama M.Y. (2009). Sugarcane Genes Associated with Sucrose Content. BMC Genom..

[18] O’Callaghan J.R., Hossain A.H.M.S., Dahab M.H., Wyseure G.C.L. (1994). SODCOM: A Solar Driven Computational Model of Crop Growth. Comput. Electron. Agric..

[19] Balsalobre T.W.A., Mancini M.C., da Pereira G.S., Anoni C.O., Barreto F.Z., Hoffmann H.P., de Souza A.P., Garcia A.A.F., Carneiro M.S. (2016). Mixed Modeling of Yield Components and Brown Rust Resistance in Sugarcane Families. Agron. J..

[20] (2006). Consecana Manual de Instruções. http://www.oricana.com.br/novosite/manual_consecana.pdf.

[21] Dubois M., Gilles K.A., Hamilton J.K., Rebers P.A., Smith F. (1956). Colorimetric Method for Determination of Sugars and Related Substances. Anal. Chem..

[22] Yang Y.H. (2002). Normalization for CDNA Microarray Data: A Robust Composite Method Addressing Single and Multiple Slide Systematic Variation. Nucleic Acids Res..

[23] Robinson M.D., McCarthy D.J., Smyth G.K. (2010). EdgeR: A Bioconductor Package for Differential Expression Analysis of Digital Gene Expression Data. Bioinformatics.

[24] Ritchie M.E., Phipson B., Wu D., Hu Y., Law C.W., Shi W., Smyth G.K. (2015). Limma Powers Differential Expression Analyses for RNA-Sequencing and Microarray Studies. Nucleic Acids Res..

[25] Alexa A., Rahnenfuhrer J. (2019). TopGO: Enrichment Analysis for Gene Ontology, Version 2.3.6 R Package. https://bioconductor.org/packages/release/bioc/html/topGO.html.

[26] Salem M.A., Jüppner J., Bajdzienko K., Giavalisco P. (2016). Protocol: A Fast, Comprehensive and Reproducible One-Step Extraction Method for the Rapid Preparation of Polar and Semi-Polar Metabolites, Lipids, Proteins, Starch and Cell Wall Polymers from a Single Sample. Plant Methods.

[27] Smith C.A., Want E.J., O’Maille G., Abagyan R., Siuzdak G. (2006). XCMS: Processing Mass Spectrometry Data for Metabolite Profiling Using Nonlinear Peak Alignment, Matching, and Identification. Anal. Chem..

[28] Kuhl C., Tautenhahn R., Böttcher C., Larson T.R., Neumann S. (2012). CAMERA: An Integrated Strategy for Compound Spectra Extraction and Annotation of Liquid Chromatography/Mass Spectrometry Data Sets. Anal. Chem..

[29] Silva R.R., Jourdan F., Salvanha D.M., Letisse F., Jamin E.L., Guidetti-Gonzalez S., Labate C.A., Vêncio R.Z.N. (2014). ProbMetab: An R Package for Bayesian Probabilistic Annotation of LC–MS-Based Metabolomics. Bioinformatics.

[30] Aggio R.B.M., Ruggiero K., Villas-Bôas S.G. (2010). Pathway Activity Profiling (PAPi): From the Metabolite Profile to the Metabolic Pathway Activity. Bioinformatics.

[31] Chong J., Soufan O., Li C., Caraus I., Li S., Bourque G., Wishart D.S., Xia J. (2018). MetaboAnalyst 4.0: Towards More Transparent and Integrative Metabolomics Analysis. Nucleic Acids Res..

[32] Xia J., Psychogios N., Young N., Wishart D.S. (2009). MetaboAnalyst: A Web Server for Metabolomic Data Analysis and Interpretation. Nucleic Acids Res..

[33] Argelaguet R., Velten B., Arnol D., Dietrich S., Zenz T., Marioni J.C., Buettner F., Huber W., Stegle O. (2018). Multi-Omics Factor Analysis—a Framework for Unsupervised Integration of Multi-Omics Data Sets. Mol. Syst. Biol..

[34] Conesa A., Götz S., García-Gómez J.M., Terol J., Talón M., Robles M. (2005). Blast2GO: A Universal Tool for Annotation, Visualization and Analysis in Functional Genomics Research. Bioinformatics.

[35] Ferreira E.B., Cavalcanti P.P., Nogueira D.A. (2014). ExpDes: An R Package for ANOVA and Experimental Designs. AM.

[36] Lembke C.G., Nishiyama M.Y., Sato P.M., de Andrade R.F., Souza G.M. (2012). Identification of Sense and Antisense Transcripts Regulated by Drought in Sugarcane. Plant Mol. Biol..

[37] Xia J., Wishart D.S. (2011). Metabolomic Data Processing, Analysis, and Interpretation Using MetaboAnalyst. Curr. Protoc. Bioinform..

[38] Brieger F.O. (1968). Início Da Safra. Como Determinar a Maturação. Bol. Inf. Copereste.

[39] Wagih M.E., Ala A., Musa Y. (2004). Evaluation of Sugarcane Varieties for Maturity Earliness and Selection for Efficient Sugar Accumulation. Sugar Tech.

[40] Prabu G., Kawar P.G., Pagariya M.C., Prasad D.T. (2011). Identification of Water Deficit Stress Upregulated Genes in Sugarcane. Plant Mol. Biol. Rep..

[41] Ebrahim M.K., Zingsheim O., El-Shourbagy M.N., Moore P.H., Komor E. (1998). Growth and Sugar Storage in Sugarcane Grown at Temperatures below and above Optimum. J. Plant Physiol..

[42] Dominy C., Haynes R., van Antwerpen R. (2002). Loss of Soil Organic Matter and Related Soil Properties under Long-Term Sugarcane Production on Two Contrasting Soils. Biol. Fertil. Soils.

[43] Robertson M.J., Wood A.W., Muchow R.C. (1996). Growth of Sugarcane under High Input Conditions in Tropical Australia. I. Radiation Use, Biomass Accumulation and Partitioning. Field Crop. Res..

[44] de Medeiros Barbosa V.C.F. (2015). Sugarcane.

[45] Carr M.K.V., Knox J.W. (2011). The Water Relations and Irrigation Requirements of Sugar Cane (*Saccharum officinarum*): A Review. Exp. Agric..

[46] Cardozo N.P., Sentelhas P.C., Panosso A.R., Palhares A.L., Ide B.Y. (2015). Modeling Sugarcane Ripening as a Function of Accumulated Rainfall in Southern Brazil. Int. J. Biometeorol..

[47] Hu Y., Li W.-C., Xu Y.-Q., Li G.-J., Liao Y., Fu F.-L. (2009). Differential Expression of Candidate Genes for Lignin Biosynthesis under Drought Stress in Maize Leaves. J. Appl. Genet..

[48] da Silva V., da Silva B.B., Albuquerque W.G., Borges C.J.R., de Sousa I.F., Neto J.D. (2013). Crop Coefficient, Water Requirements, Yield and Water Use Efficiency of Sugarcane Growth in Brazil. Agric. Water Manage..

[49] Singels A., Donaldson R.A., Smit M.A. (2005). Improving Biomass Production and Partitioning in Sugarcane: Theory and Practice. Field Crop. Res..

[50] Marli S., Aparecida F., da Graa J.P., de Matos Pereira L., Vergnia M. (2012). Sugarcane Responses at Water Deficit Conditions. Water Stress.

[51] Hennig L. (2007). Patterns of Beauty—Omics Meets Plant Development. Trends Plant Sci..

[52] Bottcher A., Cesarino I., Brombini dos Santos A., Vicentini R., Mayer J.L.S., Vanholme R., Morreel K., Goeminne G., Moura J.C.M.S., Nobile P.M. (2013). Lignification in Sugarcane: Biochemical Characterization, Gene Discovery, and Expression Analysis in Two Genotypes Contrasting for Lignin Content. Plant Physiol..

[53] Kurosaki F., Vallisuta O. (2012). Induction and Activation of Plant Secondary Metabolism by External Stimuli. Drug Discovery Research in Pharmacognosy.

[54] Tejera N., Ortega E., Rodes R., Lluch C. (2006). Nitrogen Compounds in the Apoplastic Sap of Sugarcane Stem: Some Implications in the Association with Endophytes. J. Plant Physiol..

[55] Guerzoni J.T.S., Belintani N.G., Moreira R.M.P., Hoshino A.A., Domingues D.S., Filho J.C.B., Vieira L.G.E. (2014). Stress-Induced Δ1-Pyrroline-5-Carboxylate Synthetase (P5CS) Gene Confers Tolerance to Salt Stress in Transgenic Sugarcane. Acta Physiol. Plant.

[56] Dvořáková L., Srba M., Opatrny Z., Fischer L. (2012). Hybrid Proline-Rich Proteins: Novel Players in Plant Cell Elongation?. Ann. Bot..

[57] Mattioli R., Costantino P., Trovato M. (2009). Proline Accumulation in Plants: Not Only Stress. Plant Signal. Behav..

[58] dos Santos A.B., Bottcher A., Vicentini R., Sampaio Mayer J.L., Kiyota E., Landell M.A.G., Creste S., Mazzafera P. (2015). Lignin Biosynthesis in Sugarcane Is Affected by Low Temperature. Environ. Exp. Bot..

[59] Glassop D., Roessner U., Bacic A., Bonnett G.D. (2007). Changes in the Sugarcane Metabolome with Stem Development. Are They Related to Sucrose Accumulation?. Plant. Cell Physiol..

[60] Bosch S., Rohwer J.M., Botha F.C. (2003). The Sugarcane Metabolome. Proc. S. Afr. Sug. Technol. Ass..

[61] Ferreira D.A., Martins M.C.M., Cheavegatti-Gianotto A., Carneiro M.S., Amadeu R.R., Aricetti J.A., Wolf L.D., Hoffmann H.P., de Abreu L.G.F., Caldana C. (2018). Metabolite Profiles of Sugarcane Culm Reveal the Relationship Among Metabolism and Axillary Bud Outgrowth in Genetically Related Sugarcane Commercial Cultivars. Front. Plant Sci..

[62] Chen D., Shao Q., Yin L., Younis A., Zheng B. (2018). Polyamine Function in Plants: Metabolism, Regulation on Development, and Roles in Abiotic Stress Responses. Front. Plant Sci..

[63] Luo J., Fuell C., Parr A., Hill L., Bailey P., Elliott K., Fairhurst S.A., Martin C., Michael A.J. (2009). A Novel Polyamine Acyltransferase Responsible for the Accumulation of Spermidine Conjugates in *Arabidopsis* Seed. Plant Cell.

[64] Martin-Tanguy J. (1997). Conjugated Polyamines and Reproductive Development: Biochemical, Molecular and Physiological Approaches. Physiol. Plant.

[65] Nkomo M., Gokul A., Keyster M., Klein A. (2019). Exogenous P-Coumaric Acid Improves *Salvia hispanica* L. Seedling Shoot Growth. Plants.

[66] Schenck C.A., Maeda H.A. (2018). Tyrosine Biosynthesis, Metabolism, and Catabolism in Plants. Phytochemistry.

[67] Wittstock U., Kliebenstein D.J., Lambrix V., Reichelt M., Gershenzon J. (2003). Chapter Five Glucosinolate Hydrolysis and Its Impact on Generalist and Specialist Insect Herbivores. Recent Advances in Phytochemistry.

[68] Rongai D., Cerato C., Lazzeri L. (2009). A Natural Fungicide for the Control of Erysiphe Betae and Erysiphe Cichoracearum. Eur. J. Plant Pathol..

[69] Borges A., Abreu A.C., Ferreira C., Saavedra M.J., Simões L.C., Simões M. (2015). Antibacterial Activity and Mode of Action of Selected Glucosinolate Hydrolysis Products against Bacterial Pathogens. J. Food Sci. Technol..

[70] Oliveira R.D.L., Dhingra O.D., Lima A.O., Jham G.N., Berhow M.A., Holloway R.K., Vaughn S.F. (2011). Glucosinolate Content and Nematicidal Activity of Brazilian Wild Mustard Tissues against Meloidogyne Incognita in Tomato. Plant Soil.

[71] Halkier B.A., Gershenzon J. (2006). BIOLOGY AND BIOCHEMISTRY OF GLUCOSINOLATES. Annu. Rev. Plant Biol..

[72] Dinkova-Kostova A.T., Kostov R.V. (2012). Glucosinolates and Isothiocyanates in Health and Disease. Trends Mol. Med..

[73] Johnson I.T. (2002). Glucosinolates: Bioavailability and Importance to Health. Int. J. Vitam. Nutr. Res..

[74] Claros Cuadrado J.L., Pinillos E.O., Tito R., Mirones C.S., Gamarra Mendoza N.N. (2019). Insecticidal Properties of Capsaicinoids and Glucosinolates Extracted from Capsicum Chinense and Tropaeolum Tuberosum. Insects.

[75] Wittstock U., Halkier B.A. (2002). Glucosinolate Research in the Arabidopsis Era. Trends Plant Sci..

[76] Das S.K., Mahanta S., Tanti B., Tag H., Hui P.K. (2021). Identification of Phytocompounds from Houttuynia Cordata Thunb. as Potential Inhibitors for SARS-CoV-2 Replication Proteins through GC–MS/LC–MS Characterization, Molecular Docking and Molecular Dynamics Simulation. Mol. Divers.

[77] Kandeel M., Abdelrahman A.H.M., Oh-Hashi K., Ibrahim A., Venugopala K.N., Morsy M.A., Ibrahim M.A.A. (2021). Repurposing of FDA-Approved Antivirals, Antibiotics, Anthelmintics, Antioxidants, and Cell Protectives against SARS-CoV-2 Papain-like Protease. J. Biomol. Struct. Dyn..

[78] Wang J., Zhang X., Omarini A.B., Li B. (2020). Virtual Screening for Functional Foods against the Main Protease of SARS-CoV-2. J. Food Biochem..

[79] Chikhale R.V., Gupta V.K., Eldesoky G.E., Wabaidur S.M., Patil S.A., Islam M.A. (2021). Identification of Potential Anti-TMPRSS2 Natural Products through Homology Modelling, Virtual Screening and Molecular Dynamics Simulation Studies. J. Biomol. Struct. Dyn..

[80] Gao W., Sun H.-X., Xiao H., Cui G., Hillwig M.L., Jackson A., Wang X., Shen Y., Zhao N., Zhang L. (2014). Combining Metabolomics and Transcriptomics to Characterize Tanshinone Biosynthesis in Salvia Miltiorrhiza. BMC Genom..

[81] Shin T.H., Lee D.Y., Lee H.-S., Park H.J., Jin M.S., Paik M.-J., Manavalan B., Mo J.-S., Lee G. (2018). Integration of Metabolomics and Transcriptomics in Nanotoxicity Studies. BMB Rep..

[82] Udhane S.S., Legeza B., Marti N., Hertig D., Diserens G., Nuoffer J.-M., Vermathen P., Flück C.E. (2017). Combined Transcriptome and Metabolome Analyses of Metformin Effects Reveal Novel Links between Metabolic Networks in Steroidogenic Systems. Sci. Rep..

[83] Izawa T., Mihara M., Suzuki Y., Gupta M., Itoh H., Nagano A.J., Motoyama R., Sawada Y., Yano M., Hirai M.Y. (2011). Os-*GIGANTEA* Confers Robust Diurnal Rhythms on the Global Transcriptome of Rice in the Field. Plant Cell.

[84] Kogel K.-H., Voll L.M., Schafer P., Jansen C., Wu Y., Langen G., Imani J., Hofmann J., Schmiedl A., Sonnewald S. (2010). Transcriptome and Metabolome Profiling of Field-Grown Transgenic Barley Lack Induced Differences but Show Cultivar-Specific Variances. Proc. Natl. Acad. Sci. USA.

[85] Diniz A.L., da Silva D.I.R., Lembke C.G., Costa M.D.-B.L., ten-Caten F., Li F., Vilela R.D., Menossi M., Ware D., Endres L. (2020). Amino Acid and Carbohydrate Metabolism Are Coordinated to Maintain Energetic Balance during Drought in Sugarcane. Int. J. Mol. Sci..

[86] Hildebrandt T.M. (2018). Synthesis versus Degradation: Directions of Amino Acid Metabolism during Arabidopsis Abiotic Stress Response. Plant Mol. Biol..

[87] Obata T., Fernie A.R. (2012). The Use of Metabolomics to Dissect Plant Responses to Abiotic Stresses. Cell. Mol. Life Sci..

[88] Dare A.P., Yauk Y.-K., Tomes S., McGhie T.K., Rebstock R.S., Cooney J.M., Atkinson R.G. (2017). Silencing a Phloretin-Specific Glycosyltransferase Perturbs Both General Phenylpropanoid Biosynthesis and Plant Development. Plant J..

[89] Ferrandino A., Lovisolo C. (2014). Abiotic Stress Effects on Grapevine (*Vitis vinifera* L.): Focus on Abscisic Acid-Mediated Consequences on Secondary Metabolism and Berry Quality. Environ. Exp. Bot..

[90] Davin L.B., Lewis N.G., Stafford H.A., Ibrahim R.K. (1992). Phenylpropanoid Metabolism: Biosynthesis of Monolignols, Lignans and Neolignans, Lignins and Suberins. Phenolic Metabolism in Plants.

[91] Kasirajan L., Hoang N.V., Furtado A., Botha F.C., Henry R.J. (2018). Transcriptome Analysis Highlights Key Differentially Expressed Genes Involved in Cellulose and Lignin Biosynthesis of Sugarcane Genotypes Varying in Fiber Content. Sci. Rep..

[92] Nakabayashi R., Yonekura-Sakakibara K., Urano K., Suzuki M., Yamada Y., Nishizawa T., Matsuda F., Kojima M., Sakakibara H., Shinozaki K. (2014). Enhancement of Oxidative and Drought Tolerance in Arabidopsis by Overaccumulation of Antioxidant Flavonoids. Plant J..

[93] He F., Li D., Wang D., Deng M. (2016). Extraction and Purification of Quercitrin, Hyperoside, Rutin, and Afzelin from *Zanthoxylum Bungeanum* Maxim Leaves Using an Aqueous Two-Phase System: ATPS of *Z. Bungeanum* Leaves Flavonoids. J. Food Sci..

[94] Shi M., He N., Li W., Li C., Kang W. (2018). Simultaneous Determination of Myricetrin, Quercitrin and Afzelin in Leaves of Cercis Chinensis by a Fast and Effective Method of Ionic Liquid Microextraction Coupled with HPLC. Chem. Cent. J..

[95] de Barros M., Mota da Silva L., Boeing T., Somensi L.B., Cury B.J., de Moura Burci L., Santin J.R., de Andrade S.F., Monache F.D., Cechinel-Filho V. (2016). Pharmacological Reports about Gastroprotective Effects of Methanolic Extract from Leaves of Solidago Chilensis (Brazilian Arnica) and Its Components Quercitrin and Afzelin in Rodents. Naunyn-Schmiedeberg’s Arch. Pharm..

[96] Treml J., Šmejkal K. (2016). Flavonoids as Potent Scavengers of Hydroxyl Radicals: Flavonoids versus Hydroxyl Radical. Compr. Rev. Food Sci. Food Saf..

[97] Min D.-Y., Chang H.-M., Jameel H., Lucia L., Wang Z.-G. (2014). The Structure of Lignin of Corn Stover and Its Changes Induced by Mild Sodium Hydroxide Treatment. BioResources.

[98] Wang Y., Chantreau M., Sibout R., Hawkins S. (2013). Plant Cell Wall Lignification and Monolignol Metabolism. Front. Plant Sci..

[99] Bassard J.-E., Ullmann P., Bernier F., Werck-Reichhart D. (2010). Phenolamides: Bridging Polyamines to the Phenolic Metabolism. Phytochemistry.

[100] Goujon T., Sibout R., Pollet B., Maba B., Nussaume L., Bechtold N., Lu F., Ralph J., Mila I., Barrière Y. (2003). [No Title Found]. Plant Mol. Biol..

[101] Edwards M.C., Doran-Peterson J. (2012). Pectin-Rich Biomass as Feedstock for Fuel Ethanol Production. Appl. Microbiol. Biotechnol..

[102] de Souza A.P., Leite D.C.C., Pattathil S., Hahn M.G., Buckeridge M.S. (2013). Composition and Structure of Sugarcane Cell Wall Polysaccharides: Implications for Second-Generation Bioethanol Production. Bioenergy Res..

[103] Dunstan W.R. (1902). Cyanogenesis in Plants. Part II.—The Great Millet, *Sorghum vulgare*. Proc. R. Soc. Lond..

[104] de Rosa Júnior V.E., Nogueira F.T.S., Mazzafera P., Landell M.G., Arruda P. (2007). Sugarcane Dhurrin: Biosynthetic Pathway Regulation and Evolution.

[105] Hotta C.T., Nishiyama M.Y., Souza G.M. (2013). Circadian Rhythms of Sense and Antisense Transcription in Sugarcane, a Highly Polyploid Crop. PLoS ONE.

[106] Ferreira S.S., Hotta C.T., de Poelking V.G.C., Leite D.C.C., Buckeridge M.S., Loureiro M.E., Barbosa M.H.P., Carneiro M.S., Souza G.M. (2016). Co-Expression Network Analysis Reveals Transcription Factors Associated to Cell Wall Biosynthesis in Sugarcane. Plant Mol. Biol..

[107] Ohashi Y., Nakayama N., Saneoka H., Mohapatra P.K., Fujita K. (2009). Differences in the Responses of Stem Diameter and Pod Thickness to Drought Stress during the Grain Filling Stage in Soybean Plants. Acta Physiol. Plant..

[108] Okuyama L.A., Federizzi L.C., Barbosa Neto J.F. (2005). Plant Traits to Complement Selection Based on Yield Components in Wheat. Cienc. Rural.

[109] Ferrer M.C. (2015). Morpho-Agronomic Characterization and Evaluation for Drought Tolerance of 50 Selected Philippine Traditional Rice (*Oryza sativa* L.) Varieties. Philipp. J. Crop. Sci..

[110] Tzin V., Galili G. (2010). New Insights into the Shikimate and Aromatic Amino Acids Biosynthesis Pathways in Plants. Mol. Plant.

[111] Agati G., Azzarello E., Pollastri S., Tattini M. (2012). Flavonoids as Antioxidants in Plants: Location and Functional Significance. Plant Sci..

[112] Rao M.J., Xu Y., Tang X., Huang Y., Liu J., Deng X., Xu Q. (2020). CsCYT75B1, a Citrus CYTOCHROME P450 Gene, Is Involved in Accumulation of Antioxidant Flavonoids and Induces Drought Tolerance in Transgenic Arabidopsis. Antioxidants.

