# Supplementary material for: Planting Season Impacts Sugarcane Stem Development, Secondary Metabolite Levels, and Natural Antisense Transcription
Source: Cells. 2021 Dec 8;10(12):3451. doi: 10.3390/cells10123451 (PMC8700069; doi:10.3390/cells10123451)
Supplement: Supplementary file 1 [file cells-10-03451-s001.zip › Cells MDPI Supplementary Figures 120321 final.pdf]

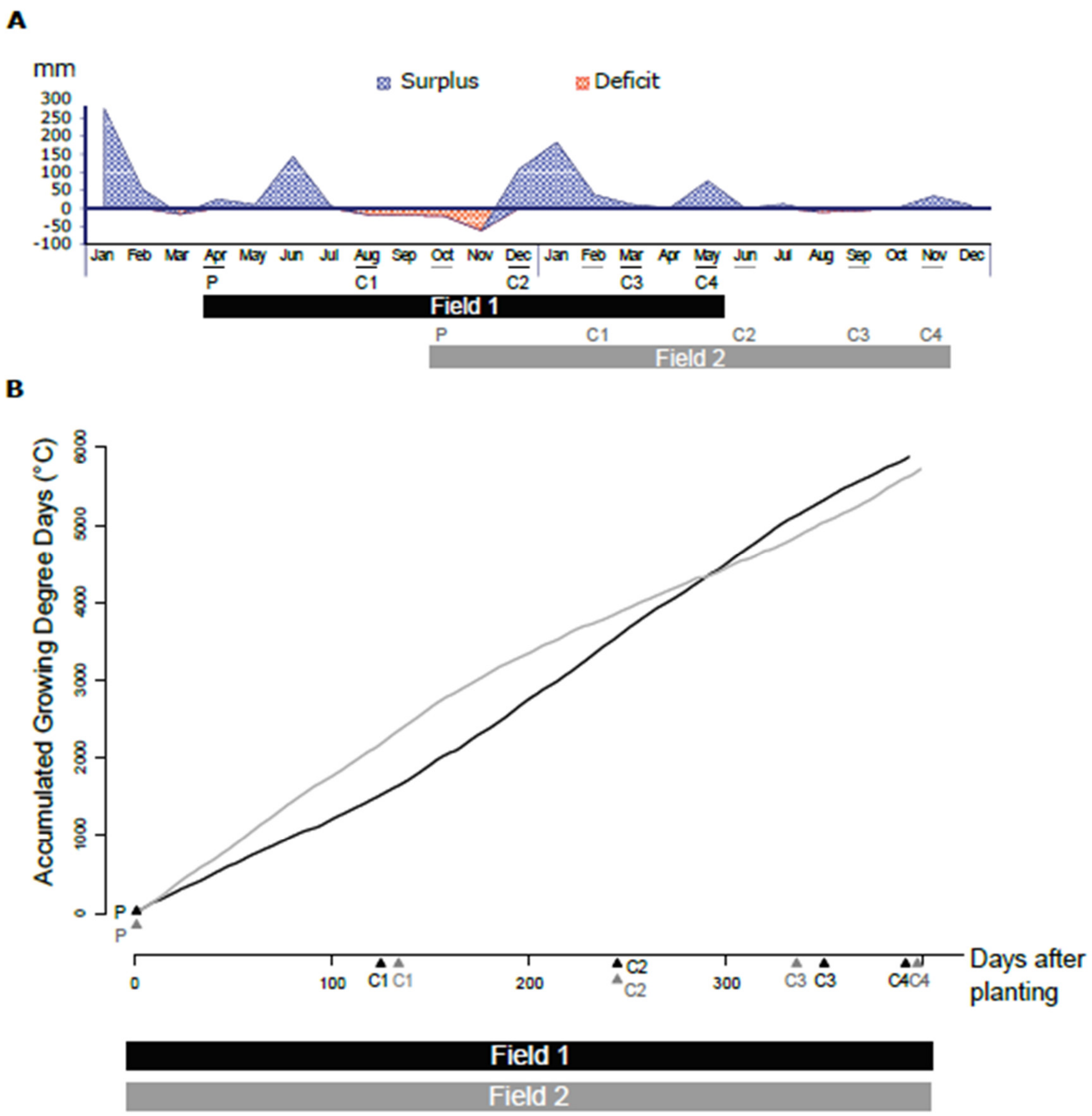

Figure S1. Soil water balance and Accumulated Growing Degree Days (AGDD) in field 1 (black bar) and field 2 (grey bar). C1, C2, C3 and C4 represent sampling points at 4, 8, 11 and 13 months after planting (P). (**A**) The Thornthwaite water balance in the soil is shown with surplus in blue and deficit in red. Inputs to the model are monthly temperature and precipitation. Data from UFSCar/CCA https://www.cca.ufscar.br/pt-br/servicos/dados-climatologicos/dados-climatologicos.xls (accessed on August 12, 2021). (**B**) AGDD values with triangles representing planting and sampling point, Black triangles in field 1 and grey triangles in field 2.

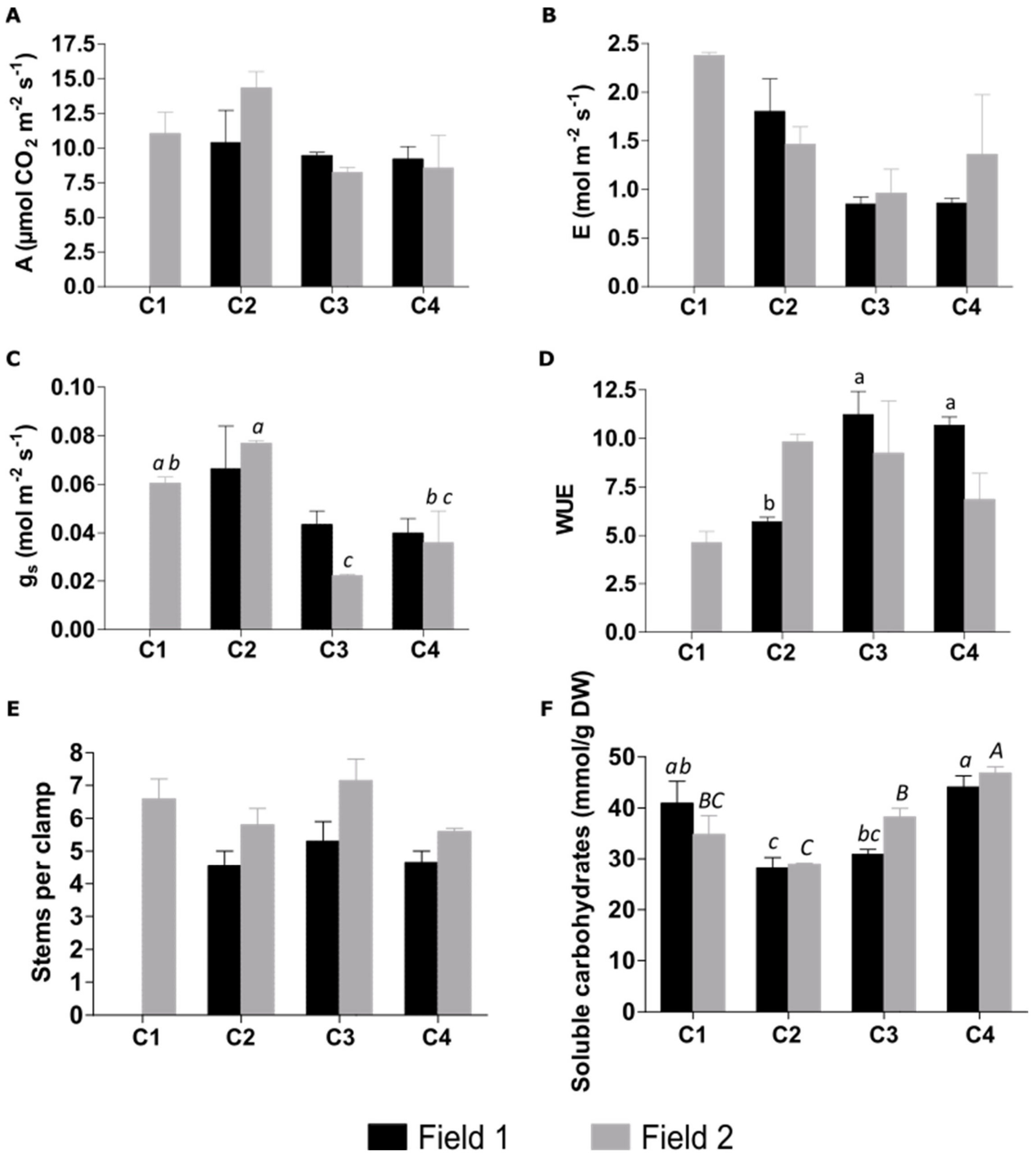

Figure S2. Instantaneous Photosynthetic rate (**A**), transpiration rate (**B**), stomatal conductance (**C**), water use efficiency (**D**), number of stems per clamp (**E**) and soluble carbohydrates in leaf (**F**) in field 1 and field 2 at 4 (C1), 8 (C2), 11 (C3) and 13 (C4)-month-old plants. Data was analyzed as split-plot scheme in balanced Completely Randomized Design (split2.crd). ANOVA and the multiple comparison test LSD were calculated. When the interaction between the two factors (field1 and field 2) was not significant, data was analyzed as Completely Randomized Design under one single factor. The analysis was done using ExpDes R Package. Regular letters and * are from split2.crd results. Bars with statistically different means are indicated by different letters (i.e. 'a' and 'b'). Bars with means that that are not statistically significant different are indicated with the same letter (i.e. 'a' and 'a') . Error bars show the standard error of the mean.

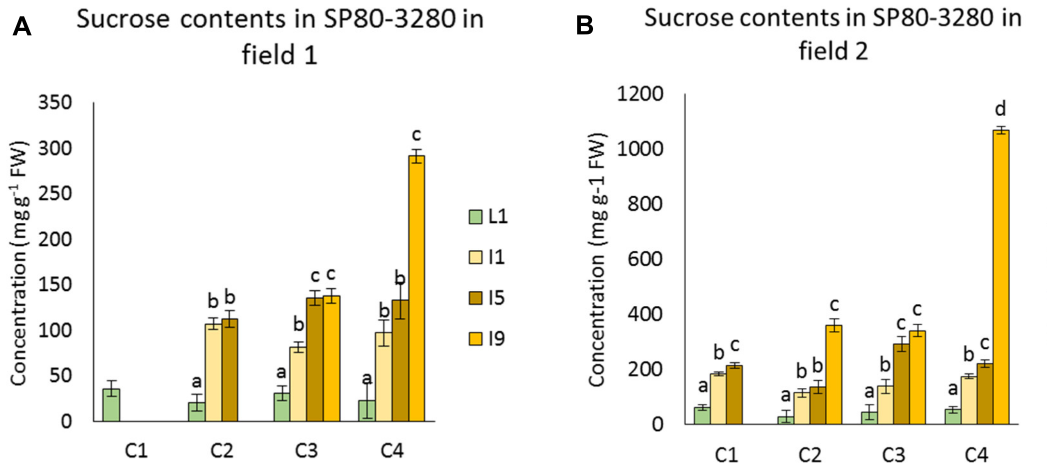

Figure S3. Sucrose contents in (**A**) field 1 and in (**B**) field 2. L1, I1, I5, and I9 refer to leaf +1 and immature, intermediate, and mature internodes. C1, C2, C3, and C4 refer to sampling points at 4, 8, 11, and 13 months after planting. The same letters (i.e. 'a' and 'a') indicate that the sucrose contents are not significantly different between the different developmental tissue within each sampling point based on ANOVA and Fisher's LSD (p-value < 0.05). Different letters (i.e. 'a' and 'b') indicate that sucrose contents are significantly different between the different developmental tissue within each sampling point based on ANOVA and Fisher's LSD (p-value < 0.05).

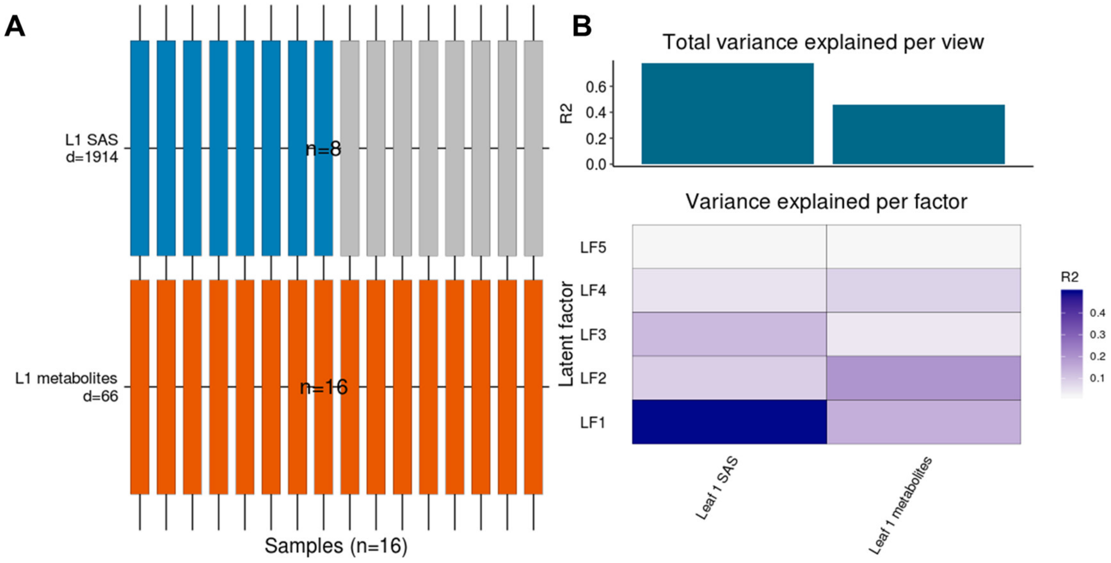

Figure S4. (**A**) Study overview and data types for leaf +1 (L1) collected from experimental field 1. Data modalities are shown in different rows (d = number of features) and samples (n) in columns, with missing samples shown using grey bars. (**B**) Proportion of total variance explained (R^2) by individual data modalities (view) and the cumulative proportion of total variance explained per factor.

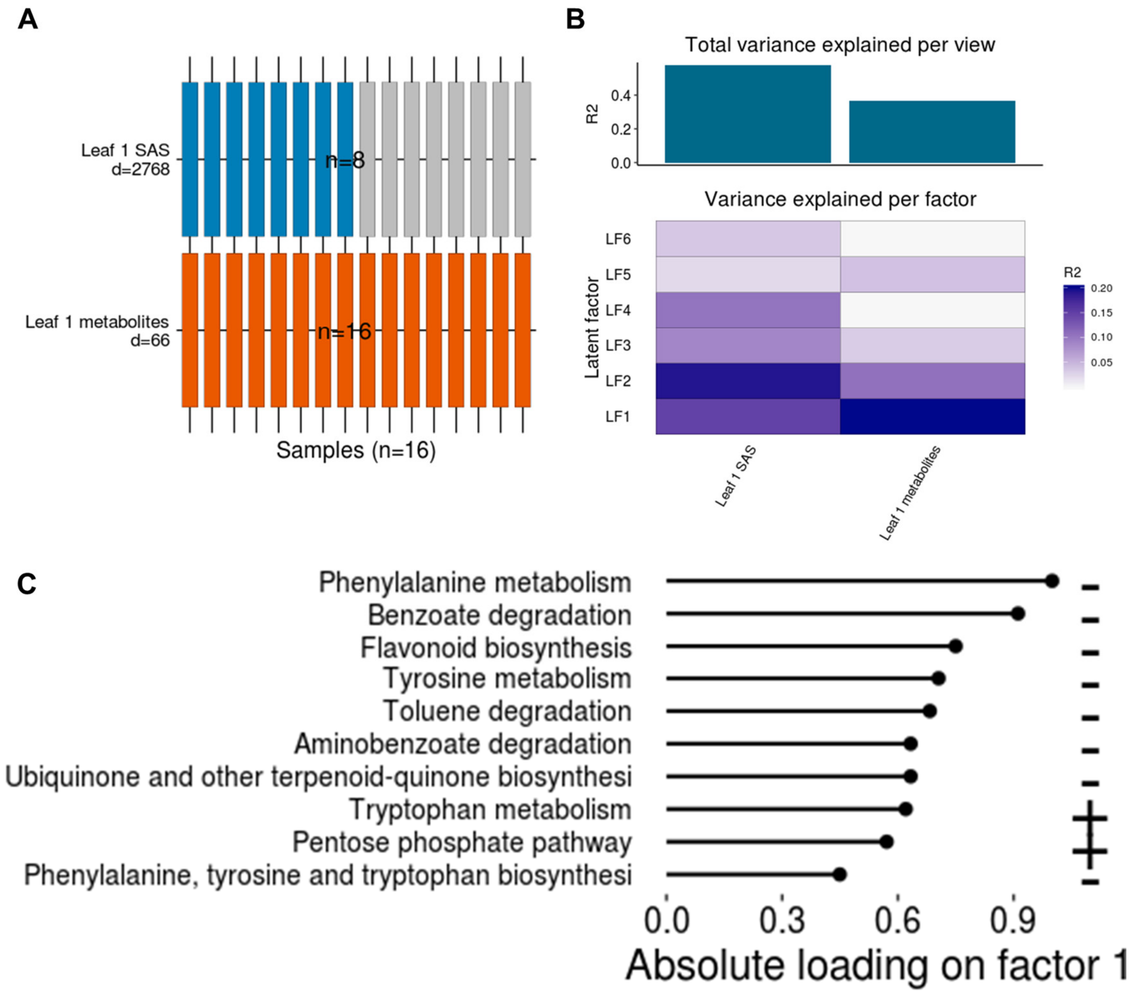

Figure S5. (**A**) Study overview and data types for leaf +1 (L1) collected from experimental field 2. Data modalities are shown in different rows (d = number of features) and samples (n) in columns, with missing samples shown using grey bars. (**B**) Proportion of total variance explained (R^2) by individual data modalities (views) and the cumulative proportion of total variance explained per factor. (**C**) Absolute loadings of the top features of Latent Factors 1.

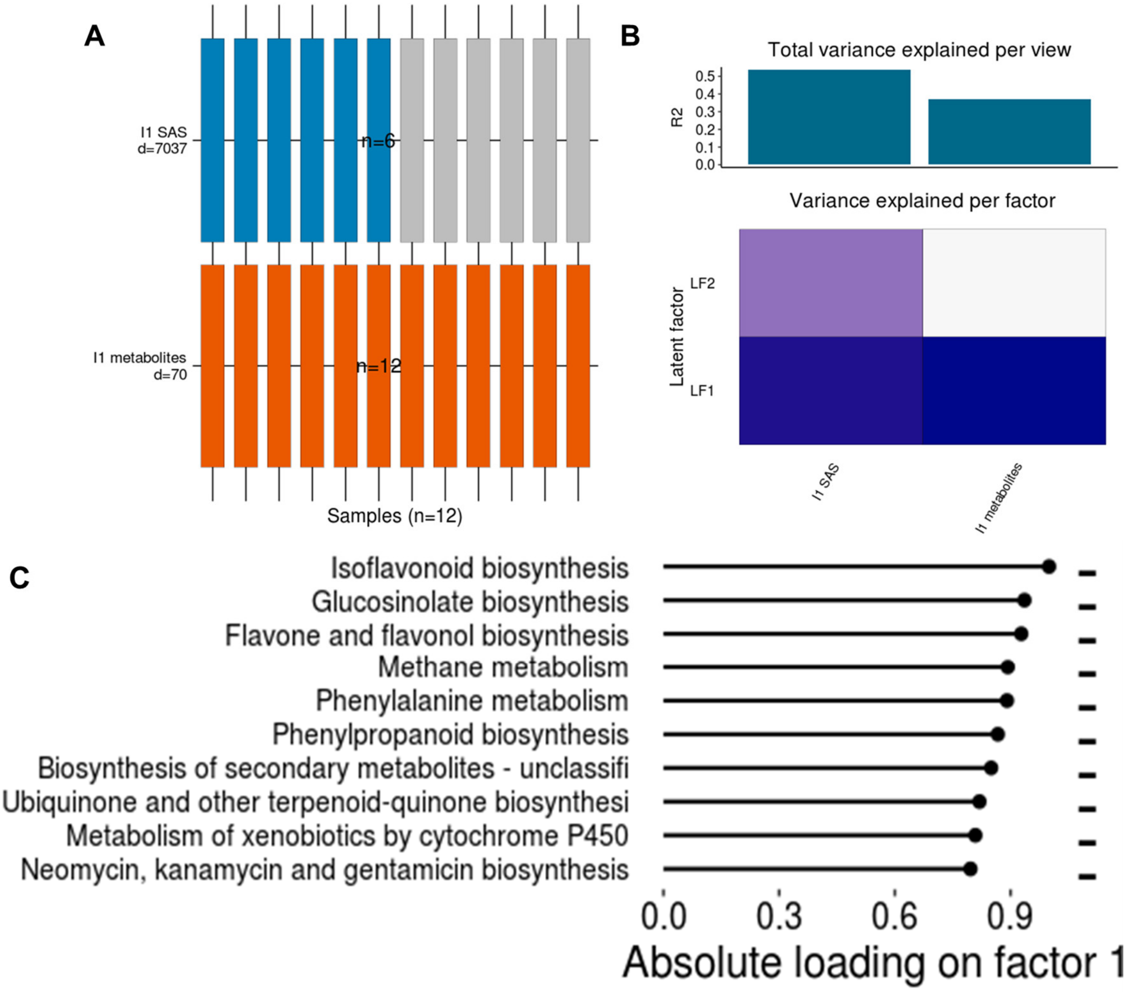

Figure S6. (**A**) Study overview and data types for immature internode (I1) collected from experimental field 1. Data modalities are shown in different rows (d = number of features) and samples (n) in columns, with missing samples shown using grey bars. (**B**) Proportion of total variance explained (R^2) by individual data modalities (views) and the cumulative proportion of total variance explained per factor. (**C**) Absolute loadings of the top features of Latent Factor 1.

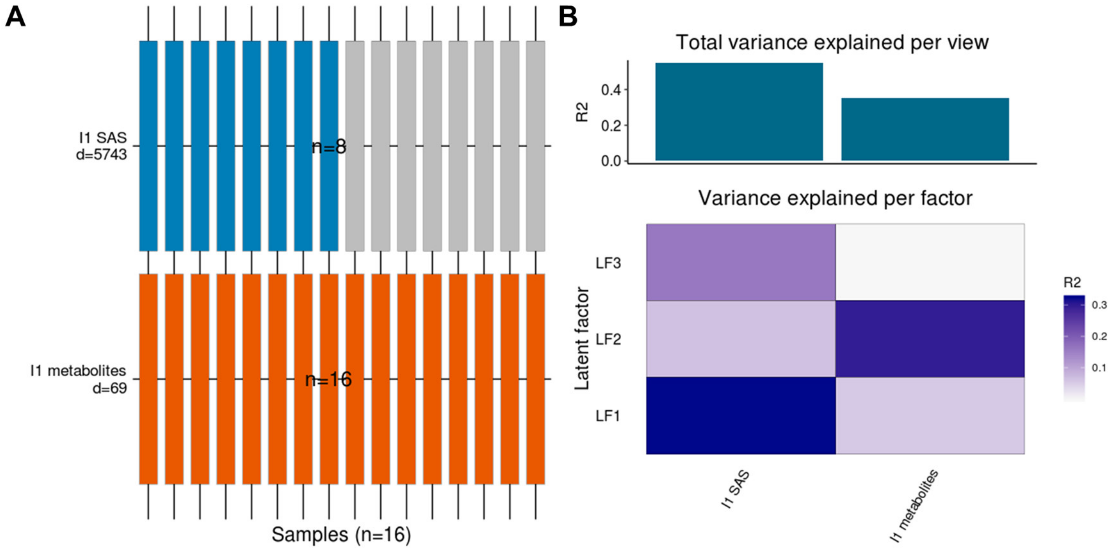

C

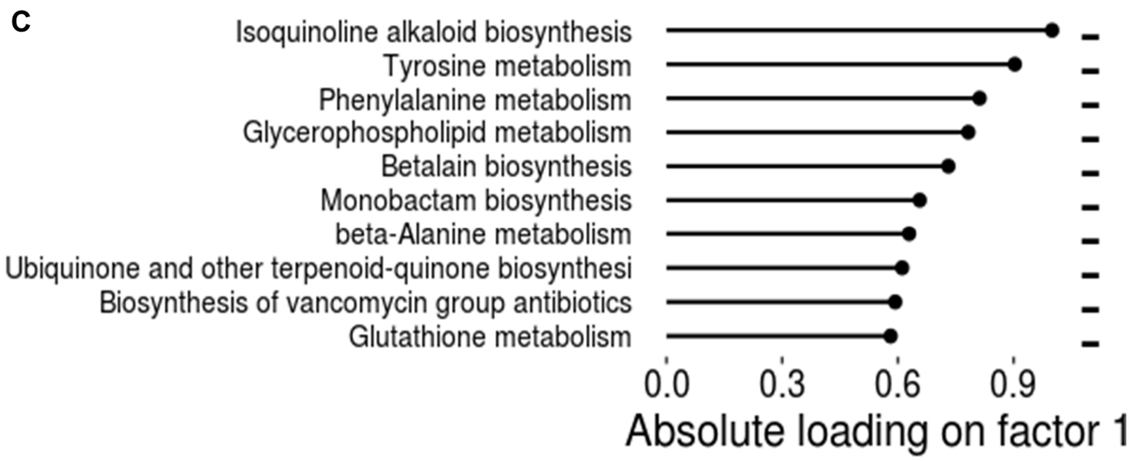

Figure S7. (**A**) Study overview and data types for immature internode (I1) collected from experimental field 2. Data modalities are shown in different rows (d = number of features) and samples (n) in columns, with missing samples shown using grey bars. (**B**) Proportion of total variance explained (R^2) by individual data modalities (views) and the cumulative proportion of total variance explained factor (**C**). Absolute loadings of the top features of Latent Factors 1.

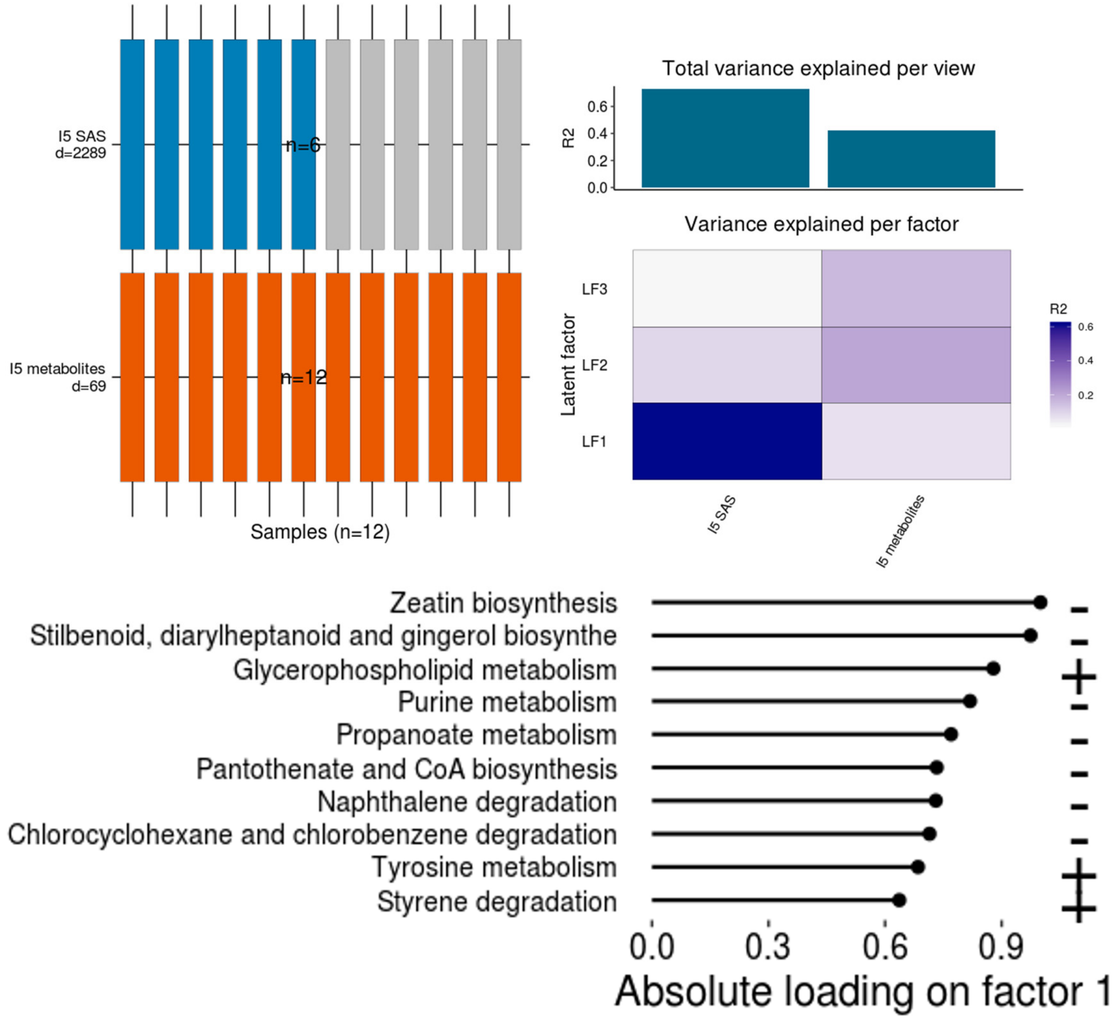

Figure S8. (A) Study overview and data types for the intermediate internodes (I5) collected from experimental field 1. Data modalities are shown in different rows (d = number of features) and samples (n) in columns, with missing samples shown using grey bars. (B) Proportion of total variance explained (R^2) by

individual data modalities (views) and the cumulative proportion of total variance explained. (C) Absolute loadings of the top features of Latent Factors 1. No correlations or shared variance between the metabolomics and transcriptomics data modalities could be identified by MOFA in samples from the I5 collected from experimental field 2.

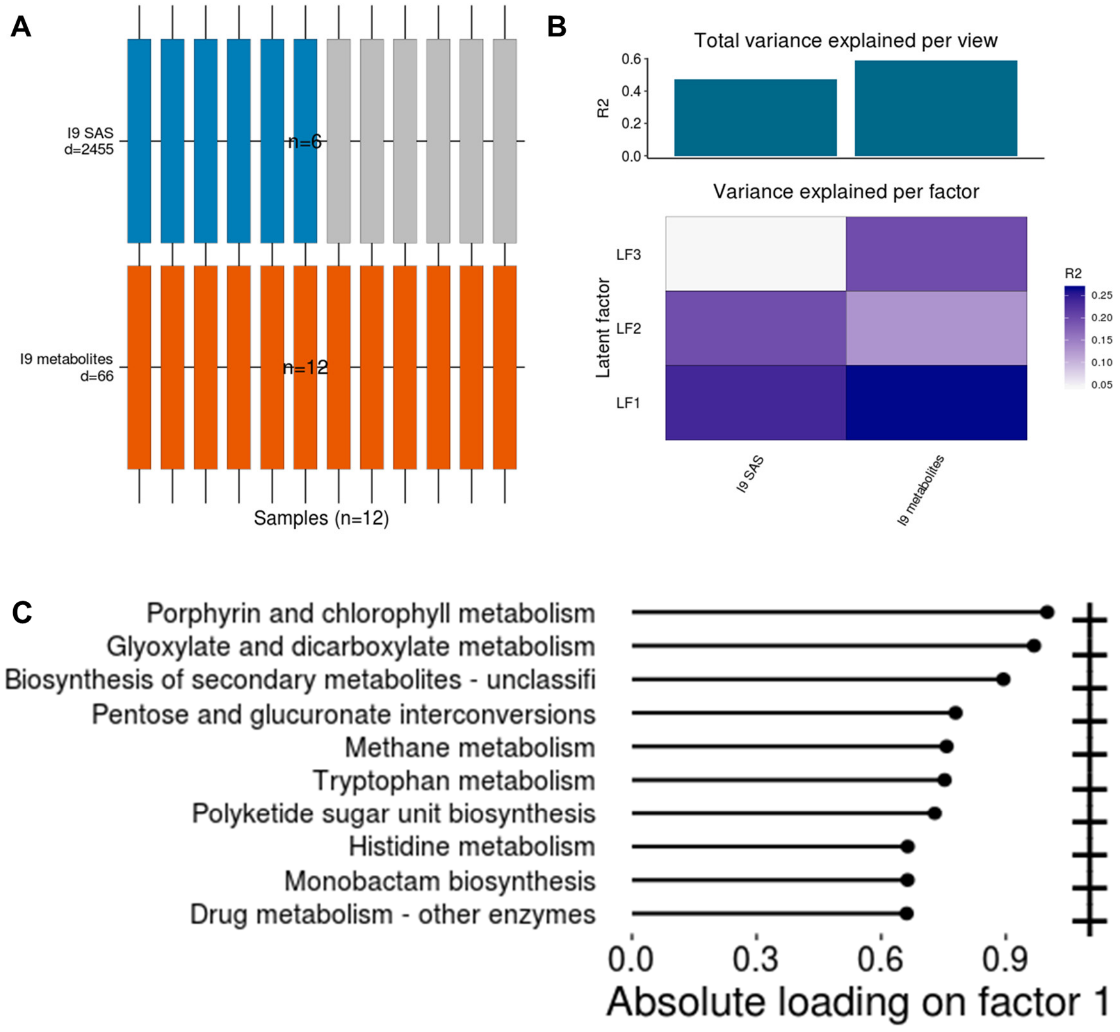

Figure S9. (**A**) Study overview and data types for mature internodes (I9) collected from experimental field 2. No correlations or shared variance between the metabolomics and transcriptomics data modalities could be identified by MOFA in I9 tissue collected from experimental field 1. Data modalities are shown in different rows (d = number of features) and samples (n) in columns, with missing samples shown using grey bars. (**B**) Proportion of total variance explained (R^2) by individual data modalities (views) and the cumulative proportion of total variance explained. (**C**) Absolute loadings of the top features of Latent Factors 1.

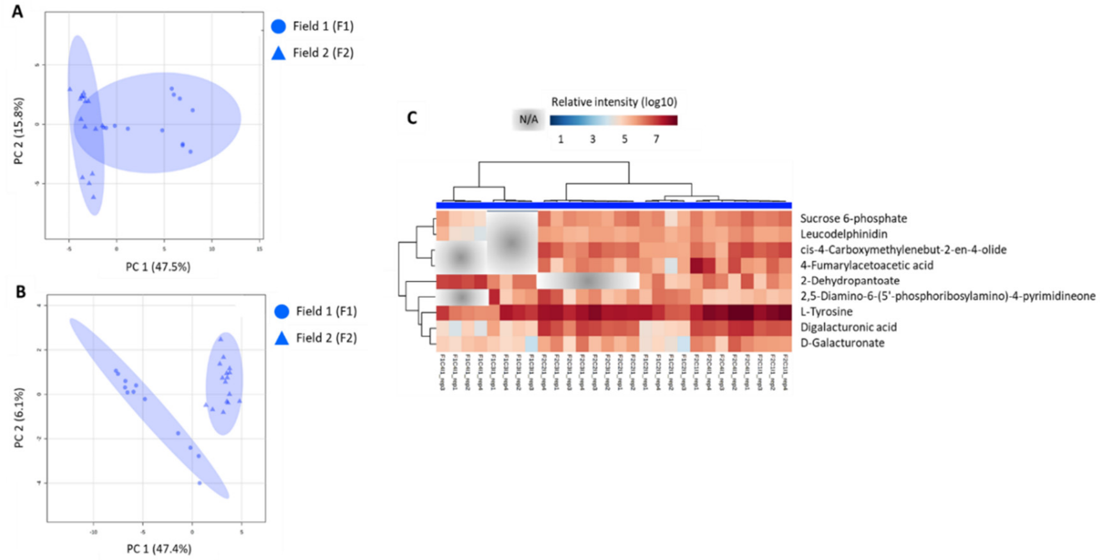

Figure S10. Discriminant models for the immature internode (I1) between fields. Discriminant models between field comparisons generated from the normalized and log10 transformed metabolomics data from the I1 tissues, combining sampling points (C1, C2, C3 and C4) and experimental fields (F1 and F2). (**A**) PCA and (**B**) PLS-DA models for I1 (component 1 Q2 = 0.70928, R2 = 0.75463 and component 2, Q2 = 0.8062, R2 = 0.91727); (**C**) Heatmap representation of the 9 main metabolites responsible for the separations (VIP scores ≥ 1.0 from PLS-DA analysis) and NA refers to non-detected metabolites.

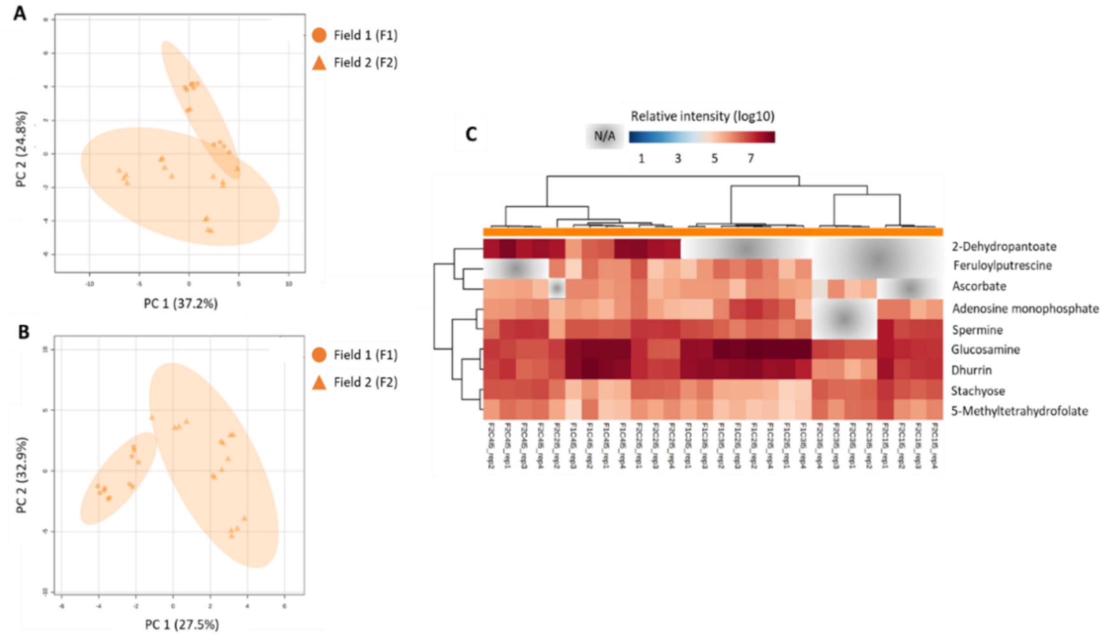

Figure S11. Discriminant models for the intermediate internode (I5) between fields. Discriminant models for between field comparisons generated from the normalized and log10 transformed metabolomics data from the I5 tissues, combining sampling points (C1, C2, C3 and C4) and experimental fields (F1 and F2). (**A**) PCA and (**B**) PLS-DA models for I5 (component 1 Q2= 0.7642, R2 = 0.82433 and component 2 Q2 = 0.78814, R2= 0.861); (**C**) Heatmap representation of the 9 main metabolites responsible for the separations (VIP scores ≥ 1.0 from PLS-DA analysis) and NA refers to non-detected metabolites.

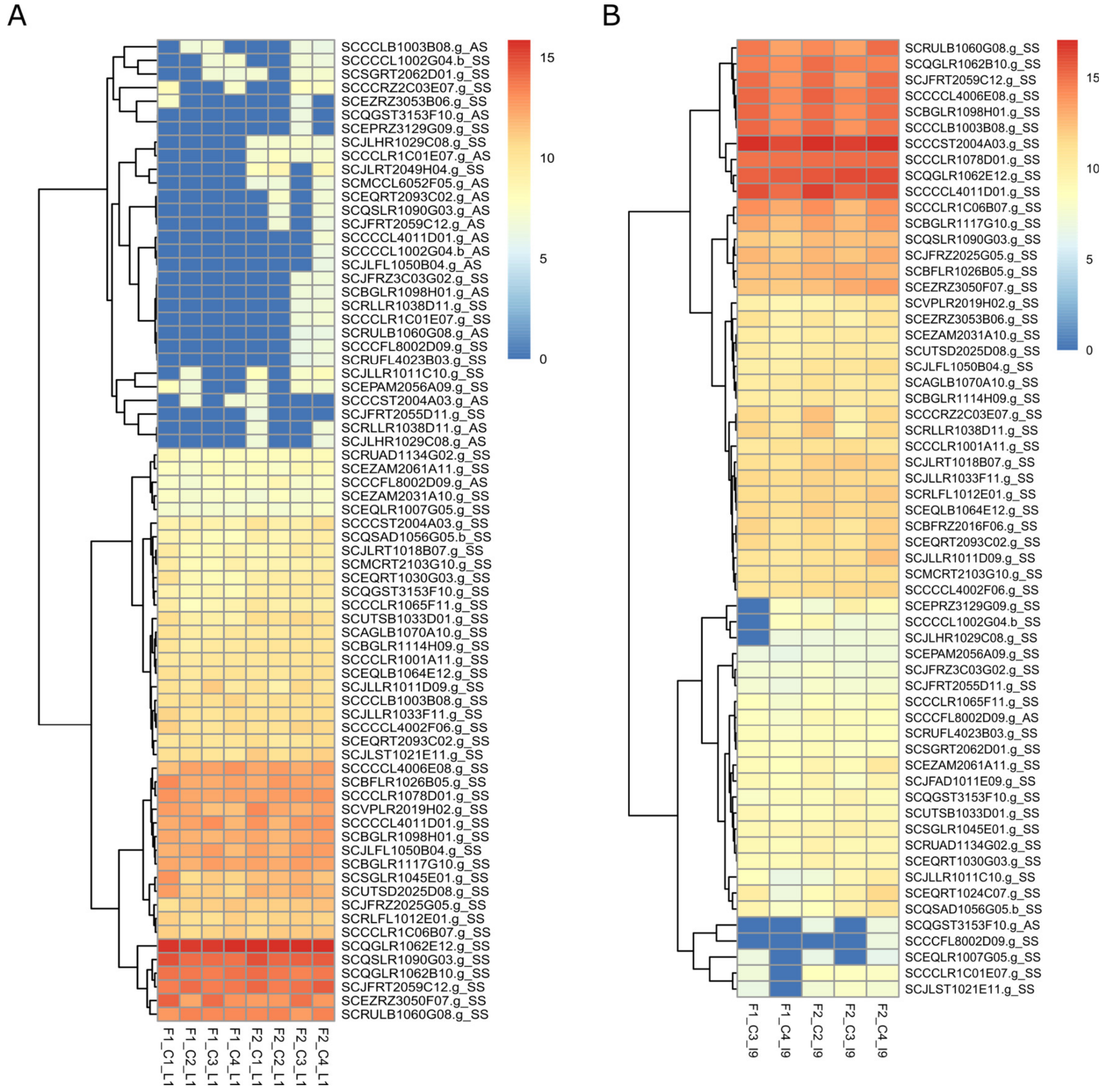

Figure S12. Natural antisense transcript expression of genes from the Phenylalanine, tyrosine and tryptophan biosynthesis pathways differs between field 1 and field 2 in leaves. Heatmap of gene expression level from genes in the (**A**) leaf +1 (L1) and in the (**B**) mature internode (I9) tissues in field 1 (F1) and field 2 (F2), and sampling points (C1, C2, C3 and C4).

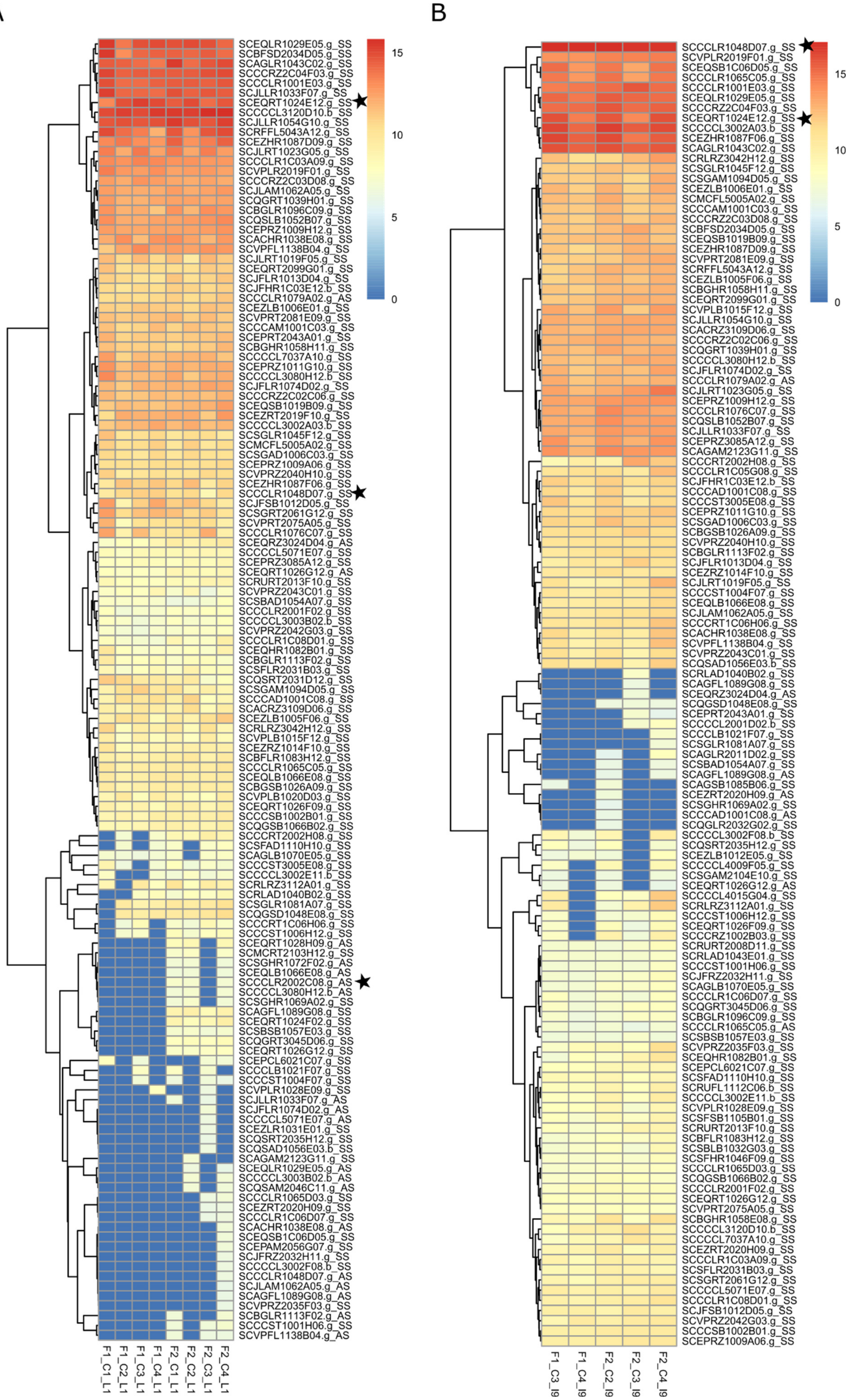
A
SCEQLR1029E05.g_SS
SCBFSD2034D05.g_SS
SCAGLR1043C02.g_SS
SCCCRZ2C04F03.g_SS
SCCCLR1001E03.g_SS
SCJLLR1033F07.g_SS
SCEQRT1024E12.g_SS
SCCCCL3120D10.b_SS
SCJLLR1054G10.g_SS
SCRFFL5043A12.g_SS
SCEZHR1087D09.g_SS
SCJLRT1023G05.g_SS
SCCCLR1C03A09.g_SS
SCVPLR2019F01.g_SS
SCCCRZ2C03D08.g_SS
SCJLAM1062A05.g_SS
SCQGRT1039H01.g_SS
SCBGLR1096C09.g_SS
SCQSLB1052B07.g_SS
SCEPRZ1009H12.g_SS
SCACHR1038E08.g_SS
SCVPFL1138B04.g_SS
SCJLRT1019F05.g_SS
SCEQRT2099G01.g_SS
SCJFLR1013D04.g_SS
SCJFHR1C03E12.b_SS
SCCCLR1079A02.g_AS
SCEZLB1006E01.g_SS
SCVPRT2081E09.g_SS
SCCCAM1001C03.g_SS
SCEPRT2043A01.g_SS
SCBGHR1058H11.g_SS
SCCCCL7037A10.g_SS
SCEPRZ1011G10.g_SS
SCCCCL3080H12.b_SS
SCJFLR1074D02.g_SS
SCCCRZ2C02C06.g_SS
SCEQSB1019B09.g_SS
SCEZRT2019F10.g_SS
SCCCCL3002A03.b_SS
SCSGLR1045F12.g_SS
SCMCFL5005A02.g_SS
SCSGAD1006C03.g_SS
SCEPRZ1009A06.g_SS
SCVPRZ2040H10.g_SS
SCEZHR1087F06.g_SS
SCCCLR1048D07.g_SS
SCJFSB1012D05.g_SS
SCSGRT2061G12.g_SS
SCVPRT2075A05.g_SS
SCCCLR1076C07.g_SS
SCEQRZ3024D04.g_AS
SCCCCL5071E07.g_SS
SCEPRZ3085A12.g_SS
SCEQRT1026G12.g_AS
SCRURT2013F10.g_SS
SCVPRZ2043C01.g_SS
SCSBAD1054A07.g_SS
SCCCLR2001F02.g_SS
SCCCCL3003B02.b_SS
SCVPRZ2042G03.g_SS
SCCCLR1C08D01.g_SS
SCEQHR1082B01.g_SS
SCBGLR1113F02.g_SS
SCSFLR2031B03.g_SS
SCQSRT2031D12.g_SS
SCSGAM1094D05.g_SS
SCCCAD1001C08.g_SS
SCACRZ3109D06.g_SS
SCEZLB1005F06.g_SS
SCRLRZ3042H12.g_SS
SCVPLB1015F12.g_SS
SCEZRZ1014F10.g_SS
SCBFLR1083H12.g_SS
SCCCLR1065C05.g_SS
SCEQLB1066E08.g_SS
SCBGSB1026A09.g_SS
SCVPLB1020D03.g_SS
SCEQRT1026F09.g_SS
SCCCSB1002B01.g_SS
SCQGSB1066B02.g_SS
SCCCRT2002H08.g_SS
SCSFAD1110H10.g_SS
SCAGLB1070E05.g_SS
SCCCST3005E08.g_SS
SCCCCL3002E11.b_SS
SCRLRZ3112A01.g_SS
SCRLAD1040B02.g_SS
SCSGLR1081A07.g_SS
SCQGSD1048E08.g_SS
SCCCRT1C06H06.g_SS
SCCCST1006H12.g_SS
SCEQRT1028H09.g_AS
SCMCRT2103H12.g_SS
SCSGHR1072F02.g_AS
SCEQLB1066E08.g_AS
SCCCLR2002C08.g_AS
SCCCCL3080H12.b_AS
SCSGHR1069A02.g_SS
SCAGFL1089G08.g_SS
SCEQRT1024F02.g_SS
SCSBSB1057E03.g_SS
SCQGRT3045D06.g_SS
SCEQRT1026G12.g_SS
SCEPCL6021C07.g_SS
SCCCLB1021F07.g_SS
SCCCST1004F07.g_SS
SCVPLR1028E09.g_SS
SCJLLR1033F07.g_AS
SCJFLR1074D02.g_AS
SCCCCL5071E07.g_AS
SCEZLR1031E01.g_SS
SCQSRT2035H12.g_SS
SCQSAD1056E03.b_SS
SCAGAM2123G11.g_SS
SCEQLR1029E05.g_AS
SCCCCL3003B02.b_AS
SCQSAM2046C11.g_AS
SCCCLR1065D03.g_SS
SCEZRT2020H09.g_SS
SCCCLR1C06D07.g_SS
SCACHR1038E08.g_AS
SCEQSB1C06D05.g_SS
SCEPAM2056G07.g_SS
SCJFRZ2032H11.g_SS
SCCCCL3002F08.b_SS
SCCCLR1048D07.g_AS
SCJLAM1062A05.g_AS
SCAGFL1089G08.g_AS
SCVPRZ2035F03.g_SS
SCBGLR1113F02.g_AS
SCCCST1001H06.g_SS
SCVPFL1138B04.g_AS
F1_C1_L1
F1_C2_L1
F1_C3_L1
F1_C4_L1
F2_C1_L1
F2_C2_L1
F2_C3_L1
F2_C4_L1
15
10
5
0
B
SCCCLR1048D07.g_SS
SCVPLR2019F01.g_SS
SCEQSB1C06D05.g_SS
SCCCLR1065C05.g_SS
SCCCLR1001E03.g_SS
SCEQLR1029E05.g_SS
SCCCRZ2C04F03.g_SS
SCEQRT1024E12.g_SS
SCCCCL3002A03.b_SS
SCEZHR1087F06.g_SS
SCAGLR1043C02.g_SS
SCRLRZ3042H12.g_SS
SCSGLR1045F12.g_SS
SCSGAM1094D05.g_SS
SCEZLB1006E01.g_SS
SCMCFL5005A02.g_SS
SCCCAM1001C03.g_SS
SCCCRZ2C03D08.g_SS
SCBFSD2034D05.g_SS
SCEQSB1019B09.g_SS
SCEZHR1087D09.g_SS
SCVPRT2081E09.g_SS
SCRFFL5043A12.g_SS
SCEZLB1005F06.g_SS
SCBGHR1058H11.g_SS
SCEQRT2099G01.g_SS
SCVPLB1015F12.g_SS
SCJLLR1054G10.g_SS
SCACRZ3109D06.g_SS
SCCCRZ2C02C06.g_SS
SCQGRT1039H01.g_SS
SCCCCL3080H12.b_SS
SCJFLR1074D02.g_SS
SCCCLR1079A02.g_AS
SCJLRT1023G05.g_SS
SCEPRZ1009H12.g_SS
SCCCLR1076C07.g_SS
SCQSLB1052B07.g_SS
SCJLLR1033F07.g_SS
SCEPRZ3085A12.g_SS
SCAGAM2123G11.g_SS
SCCCRT2002H08.g_SS
SCCCLR1C05G08.g_SS
SCJFHR1C03E12.b_SS
SCCCAD1001C08.g_SS
SCCCST3005E08.g_SS
SCEPRZ1011G10.g_SS
SCSGAD1006C03.g_SS
SCBGSB1026A09.g_SS
SCVPRZ2040H10.g_SS
SCBGLR1113F02.g_SS
SCJFLR1013D04.g_SS
SCEZRZ1014F10.g_SS
SCJLRT1019F05.g_SS
SCCCST1004F07.g_SS
SCEQLB1066E08.g_SS
SCJLAM1062A05.g_SS
SCCCRT1C06H06.g_SS
SCACHR1038E08.g_SS
SCVPFL1138B04.g_SS
SCVPRZ2043C01.g_SS
SCQSAD1056E03.b_SS
SCRLAD1040B02.g_SS
SCAGFL1089G08.g_SS
SCEQRZ3024D04.g_AS
SCQGSD1048E08.g_SS
SCEPRT2043A01.g_SS
SCCCCL2001D02.b_SS
SCCCLB1021F07.g_SS
SCSGLR1081A07.g_SS
SCAGLR2011D02.g_SS
SCSBAD1054A07.g_SS
SCAGFL1089G08.g_AS
SCAGSB1085B06.g_SS
SCEZRT2020H09.g_AS
SCSGHR1069A02.g_SS
SCCCAD1001C08.g_AS
SCQGLR2032G02.g_SS
SCCCCL3002F08.b_SS
SCQSRT2035H12.g_SS
SCEZLB1012E05.g_SS
SCCCCL4009F05.g_SS
SCSGAM2104E10.g_SS
SCEQRT1026G12.g_AS
SCCCCL4015G04.g_SS
SCRLRZ3112A01.g_SS
SCCCST1006H12.g_SS
SCEQRT1026F09.g_SS
SCCCRZ1002B03.g_SS
SCRURT2008D11.g_SS
SCRLAD1043E01.g_SS
SCCCST1001H06.g_SS
SCJFRZ2032H11.g_SS
SCAGLB1070E05.g_SS
SCCCLR1C06D07.g_SS
SCQGRT3045D06.g_SS
SCBGLR1096C09.g_SS
SCCCLR1065C05.g_AS
SCSBSB1057E03.g_SS
SCVPRZ2035F03.g_SS
SCEQHR1082B01.g_SS
SCEPCL6021C07.g_SS
SCSFAD1110H10.g_SS
SCRUFL1112C06.b_SS
SCCCCL3002E11.b_SS
SCVPLR1028E09.g_SS
SCSFSB1105B01.g_SS
SCRURT2013F10.g_SS
SCBFLR1083H12.g_SS
SCSBLB1032G03.g_SS
SCSFHR1046F09.g_SS
SCCCLR1065D03.g_SS
SCQGSB1066B02.g_SS
SCCCLR2001F02.g_SS
SCEQRT1026G12.g_SS
SCVPRT2075A05.g_SS
SCBGHR1058E08.g_SS
SCCCCL3120D10.b_SS
SCCCCL7037A10.g_SS
SCEZRT2020H09.g_SS
SCCCLR1C03A09.g_SS
SCSFLR2031B03.g_SS
SCSGRT2061G12.g_SS
SCCCCL5071E07.g_SS
SCCCLR1C08D01.g_SS
SCJFSB1012D05.g_SS
SCVPRZ2042G03.g_SS
SCCCSB1002B01.g_SS
SCEPRZ1009A06.g_SS
F1_C3_l9
F1_C4_l9
F2_C2_l9
F2_C3_l9
F2_C4_l9
15
10
5
0

Figure S13. Natural antisense transcript expression of genes from the Phenylpropanoid biosynthesis pathways differs between field 1 and field 2 in leaves. Heatmap of gene expression level from genes in the (**A**) leaf +1 (L1) and in the (**B**) mature internode (I9) tissues in field 1 (F1) and field 2 (F2), and sampling points (C1, C2, C3 and C4). The five-pointed stars indicate transcripts cited in the manuscript.

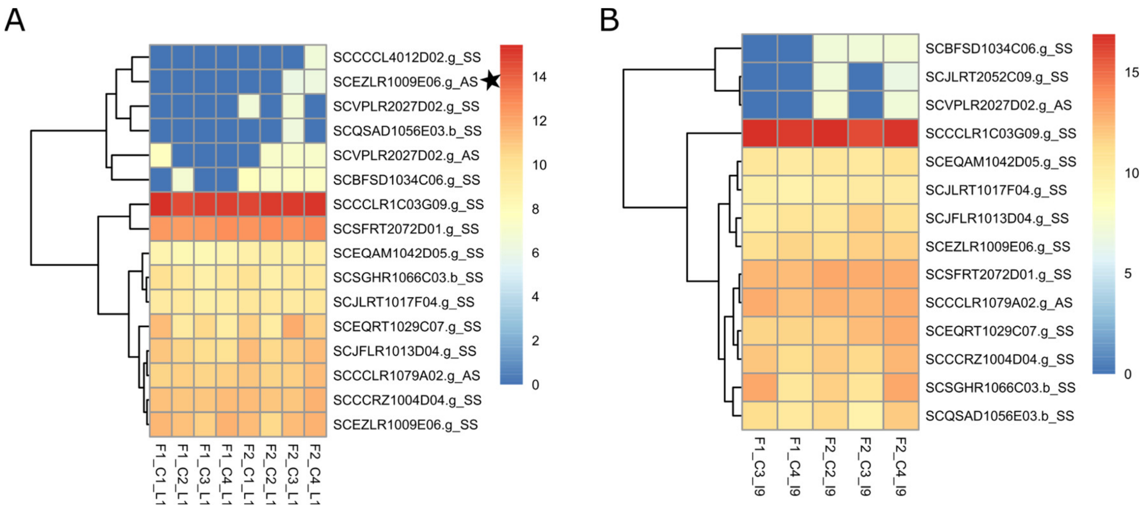

Figure S14. Gene expression of genes from the Flavonoid biosynthesis pathways in field 1 and field 2. Heatmap of gene expression level from genes in the (**A**) leaf +1 (L1) and in the (**B**) mature internode (I9) tissues in field 1 (F1) and field 2 (F2), and sampling points (C1, C2, C3 and C4). The five-pointed star indicates transcripts cited in the manuscript.
